# Evidence of a wide gap between COVID-19 in humans and animal models: a systematic review

**DOI:** 10.1186/s13054-020-03304-8

**Published:** 2020-10-06

**Authors:** Salleh N. Ehaideb, Mashan L. Abdullah, Bisher Abuyassin, Abderrezak Bouchama

**Affiliations:** Experimental Medicine Department, King Abdullah International Medical Research Center/King Saud bin Abdulaziz University for Health Sciences, King Abdulaziz Medical City, Ministry of National Guard Health Affairs, Riyadh, Saudi Arabia

**Keywords:** COVID-19, SARS-CoV-2, Animal models, Review, Non-human primate, Rodent, Hamster, Ferrets

## Abstract

**Background:**

Animal models of COVID-19 have been rapidly reported after the start of the pandemic. We aimed to assess whether the newly created models reproduce the full spectrum of human COVID-19.

**Methods:**

We searched the MEDLINE, as well as BioRxiv and MedRxiv preprint servers for original research published in English from January 1 to May 20, 2020. We used the search terms (COVID-19) OR (SARS-CoV-2) AND (animal models), (hamsters), (nonhuman primates), (macaques), (rodent), (mice), (rats), (ferrets), (rabbits), (cats), and (dogs). Inclusion criteria were the establishment of animal models of COVID-19 as an endpoint. Other inclusion criteria were assessment of prophylaxis, therapies, or vaccines, using animal models of COVID-19.

**Result:**

Thirteen peer-reviewed studies and 14 preprints met the inclusion criteria. The animals used were nonhuman primates (*n* = 13), mice (*n* = 7), ferrets (*n* = 4), hamsters (*n* = 4), and cats (*n* = 1). All animals supported high viral replication in the upper and lower respiratory tract associated with mild clinical manifestations, lung pathology, and full recovery. Older animals displayed relatively more severe illness than the younger ones. No animal models developed hypoxemic respiratory failure, multiple organ dysfunction, culminating in death. All species elicited a specific IgG antibodies response to the spike proteins, which were protective against a second exposure. Transient systemic inflammation was observed occasionally in nonhuman primates, hamsters, and mice. Notably, none of the animals unveiled a cytokine storm or coagulopathy.

**Conclusions:**

Most of the animal models of COVID-19 recapitulated mild pattern of human COVID-19 with full recovery phenotype. No severe illness associated with mortality was observed, suggesting a wide gap between COVID-19 in humans and animal models.

## Background

Coronavirus disease 2019 (COVID-19) is a febrile respiratory illness due to a novel viral pathogen severe acute respiratory syndrome–coronavirus 2 (SARS-CoV-2) [1, 2]. COVID-19 can progress to acute respiratory distress syndrome (ARDS), multiple organ dysfunction/failure (MOSD) including central nervous system alteration, acute kidney injury, cardiovascular failure, liver injury, and coagulopathy culminating in death [2–9].

SARS-CoV-2 is a beta coronavirus that binds with a high affinity to angiotensin-converting enzyme (ACE) 2 receptor and uses the transmembrane serine protease (TMPRSS) 2 as co-receptor to gain entry to cells [10–12]. ACE2 and TMPRSS2 are co-expressed in many tissues and organs, particularly the nasal epithelial cells and alveolar type II cells of the lungs, which may explain in part the easy transmission from person-to-person, and its dissemination within the body in severe and fatal cases [11–18]. Accordingly, SARS-CoV-2-induced COVID-19 has led to a pandemic that overwhelmed the capacity of most national health systems, resulting in a global health crisis [19]. So far, an estimated 11,280 million persons in 188 countries were infected, of which 531,000 died [20].

The clinical spectrum of COVID-19 is complex and has been categorized as mild, severe, and critical, representing 81, 14, and 5% [2, 3]. The mild pattern comprises patients with either no signs and symptoms or fever and radiological evidence of pneumonia [3]. The severe pattern manifests as rapidly progressive hypoxemic pneumonia involving more than half of the lung with a full recovery phenotype [2, 3]. The critical pattern consists of ARDS requiring respiratory assistance and MOSD that result in death in approximately half of the patients [2, 3, 7, 21]. Mortality was associated with host factors such as old age, comorbidities, and immune response [4].

Viral and immunopathological studies revealed distinct patterns between mild and severe or critical forms of COVID-19 [4, 5, 9, 21–27]. Both severe and critically ill patients displayed higher viral load in the upper respiratory tract than mild cases, together with delayed clearance overtime [21, 22]. Likewise, they presented with lymphopenia due to a decrease in CD4+ and CD8+ T cells, as well as T cell exhaustion accompanied by a marked inflammatory response [5, 9, 24–27]. Pro- and anti-inflammatory cytokines and chemokine concentrations were increased systemically and locally in the lung and correlated with severity [5, 9, 24]. In contrast, in the mild illness, the lymphocyte count was normal, with no or minimal inflammatory response [5, 23]. Together, these suggest that the viral load and dynamic together with the host inflammatory response may play a pathogenic role.

Clinical and post-mortem studies of fatal cases of COVID-19 demonstrated major alteration of coagulation and fibrinolysis [17, 18]. This was associated with widespread thrombosis of small and large vessels, particularly of the pulmonary circulation contributing to death in a third of patients [8, 28–33]. These observations suggest that dysregulated coagulation may be an important mechanism of COVID-19 morbidity and mortality [34].

In this context, animal models appear crucial to a better understanding of the complex biology of COVID-19. Animal models of SARS-CoV-2-induced COVID-19 have been rapidly reported since the start of the pandemic [35]. However, whether they express the full phenotype of COVID-19, particularly the severe and critical patterns associated with lethality, remains to be determined. In this systematic review, we examined whether the newly created animal models reproduce the phenotype of human COVID-19. Moreover, we examined the knowledge generated by these models of COVID-19 including viral dynamic and transmission, pathogenesis, and testing of therapy and vaccines.

## Methods

### Search strategy and selection criteria

We conducted a systematic review according to the Preferred Reporting Items for Systematic Reviews and Meta-analysis (PRISMA) statement [36] to identify studies describing the creation of an animal model of COVID-19 as an endpoint (Table 1 and Additional file 1). Additional file 1 shows the data extraction and appraisal approach as well as the selected outcome.

**Table 1:** Search strategy and selection criteria

**Fig. 1:**
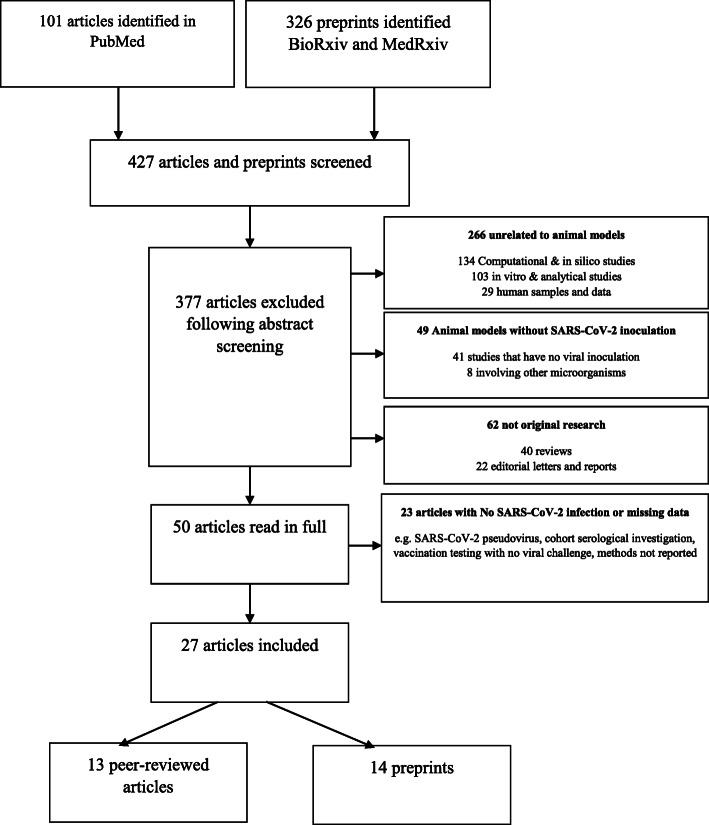
Flow diagram illustrating the process of study selection. A systematic review was conducted according to the Preferred Reporting Items for Systematic Reviews and Meta-analysis (PRISMA). *Flow diagram

We searched the MEDLINE, as well as BioRxiv and MedRxiv preprint servers for original research describing or using an animal model of SARS-CoV-2 induced COVID published in English from January 1, 2020, to May 20, 2020. We used the search terms (COVID-19) OR (SARS-CoV-2) AND, (animal models), (hamsters), (nonhuman primates), (macaques), (rodent), (mice), (rats), (ferrets), (rabbits), (cats), and (dogs). The preprint servers were included in the search as the field of COVID-19 is developing quickly. Inclusion criteria were the establishment of animal models of COVID-19 as an endpoint. Other inclusion criteria were assessment of prophylaxis, therapies, or vaccines, using animal models of COVID-19. Exclusion criteria consisted of reviews, non-original articles, and unrelated to the COVID-19 infection or experimental animals that do not support SARS-CoV-2 replication. 101 studies and 326 preprints were screened of which 13 peer-reviewed studies and 14 preprints were included in the final analysis (Fig. 1). The variables extracted were the population type, study aim, the virus strain used, clinical response, pathology, viral replication, and host response as well as the effects of prophylaxis, drugs, or vaccines. The outcomes were organized according to species and categorized into phenotype (signs or symptoms; histopathology, time-course of the illness and outcome), viral (titer in each tissue organ, detection methods, duration of positivity), and host response (dynamic of seroconversion, inflammatory, and hemostatic markers), therapy, and vaccine (efficacy and safety)	

## Results

The systematic search identified 101 studies and 326 preprints, of which 400 articles were excluded because they were reviews, non-original articles, unrelated to the COVID-19 infection, or experimental animals that do not support SARS-CoV-2 replication such as pigs, ducks, and chickens (Fig. 1 and Additional file 2). Additional file 2 displays all the excluded studies and the rationale for their exclusion. Thirteen peer-reviewed studies and 14 preprints were included in the analysis.

The studies used nonhuman primates (*n* = 13) [37–49], mice (*n* = 7) [50–56], hamsters (*n* = 4) [56–59], ferrets (*n* = 4) [60–63], cats, and dogs (*n* = 1) [63] (Tables 2, 3, 4, and 5). Male and female, as well as young and old, were included but with no associated comorbidities. The aims were to investigate the pathogenesis of COVID-19 (*n* = 15), testing drugs and vaccines (*n* = 14), the host immune response (*n* = 6), and the virus dynamic and transmission (*n* = 4) (Tables 2, 3, 4, and 5).

**Table 2:** Summary of studies using nonhuman primate models of COVID-19

Species (ref)	Number age (gender)	Virus strain dose* (inoculation route)†	Clinical signs & observation duration (DPI) §	Viral replication‡ (DPI)	Pathology & sacrificing date (DPI)	Immune response	Seroconversion (DPI)	Outcome measures
Rhesus macaques	*n* = 8	SARS-CoV-2 nCoV-WA1–2020	Fever	Nose, oropharynx, lung	Anemia	At 1 dpi only, significant increases in IL1ra, IL6, IL10, IL15, MCP-1, MIP-1b	IgG antibody anti-spike protein (10)	Pathogenesis of COVID-19
	Adults		Weight loss	Rectum (1)	Mild to moderate, interstitial pneumonia,			
Munster et al. (2020) [37]	(M/F)	4 × 10^5^ TCID_50_ (IT, IN, PO)	Dyspnea Tachypnea		Edema			
			Piloerection		Hyaline membranes formation	At 3 dpi decrease in TGFα		
			Reduced appetite		Hyperplasia type II pneumocytes			
			Hunched posture		Swollen mediastinal lymph nodes (3, 4, 21)			
			Pale appearance					
			Dehydration (21)					
Rhesus macaques	*n* = 3	BetaCoV/Wuhan/IVDC-HB-01/2020	Weight loss	Nose, oropharynx, lung	Interstitial pneumonia	Decreased CD4+ T and CD8+ T cells in young and old.	IgG antibody anti- SARS-CoV-2 (14)	Pathogenesis of COVID-19 in aging animals
	3–5 years		Asthenia	Rectum, alveolar epithelia	Inflammation			
Yu et al. (2020) [38]	*n* = 2	1 × 10^6^ TCID_50_ (IT)	More severe in old than young (14)	Macrophages (3)	Edema			
	15 years (NA)‖			Higher replication in old than young	More severe in old than young (7)			
Rhesus macaques	*n* = 4 per group (6 vaccinated groups)	DNA vaccine**	NA‖ (14)	Lowest BAL levels of viral RNA with full-length S protein encoding vaccine	NA	Upregulation IFN-γ antipeptide spike proteins.	IgG antibody anti- SARS-CoV-2 (day14 post-vaccination)	Evaluation of candidate DNA vaccine
		IM at week 0 and week 3						
Yu et al. (2020) [39]	6–12 years (M/F)	1.1 × 10^4^ PFU (IN and IT) (day 21 post-vaccine)				S1 and RBD lower response than other variant Spike proteins**		
	*n* = 10 sham control	1.1 × 10^4^ PFU (IN and IT)		High BAL levels of viral RNA	NA	Anamnestic humoral and cellular immune responses including IFN-γ ELISPOT responses	NA	
	6–12 years (M/F)							
Rhesus macaques	*n* = 6 vaccine	2.5 × 10^10^ ChAdOx1 nCoV-19 (IM)	Tachypnea (3/6), dyspnea (2/6),	Nose, BAL (2/6)	NO	Upregulation of IFN-γ (1)	IgG antibody anti-SARS-CoV-2 spike protein (day 14 post-vaccination)	Evaluation of DNA vaccine
	M/F	SARS-CoV-2 nCoV-WA1-2020	Ruffled fur (1/6) (7)	Lung (very low), oropharynx, mediastinal, duodenum (3)				
		2.6 × 10^6^ TCID_50_ (IT, IN, PO, CJ) (day 28 post-vaccine)		No BAL subgenomic viral RNA		No difference in TNF-α, IL-2, IL-4, IL-6, and IL-10 vaccine vs. control		
Van Doremalen et al. (2020) [40]	*n* = 3 control`	Vaccinated with 2.5 × 10^10^ ChAdOx1 GFP (IM)	Tachypnea (3/3) Ruffled fur (2/3) Diarrhea (1/3) Pale appearance (1/3)	BAL, nasal swabs, lung, cervical, mediastinal lymph nodes, duodenum, urinary bladder	Interstitial pneumonia (2 of 3)	TNF-α, IL-2, IL-4, IL-6, and IL-10	NA	
					Thickening of alveolar septae			
	M/F`		Red nose (1/3)		Edema			
		SARS-CoV-2 nCoV-WA1-2020		BAL subgenomic viral RNA (3, 5)	Hyperplasia type I & II pneumocytes syncytial cells			
		2.6 × 10^6^ TCID_50_ (IT, IN, PO, CJ) (day 28 post-vaccine)			No extra pulmonary injury			
Rhesus macaques	*n* = 4 per vaccine group	PiCoVacc 6 μg/dose (high) or 3 μg/dose (low) at 0, 7, and 14 days (IM)	NA	Pharyngeal, anal, and pulmonary (3)	Mild and focal histopathological changes both lower lobes	No differences CD3+, CD4+, CD8+, TNF-α, IFN-γ, IL-2, IL-4, IL-5, IL-6 vaccine vs. control	IgG antibody anti-SARS-CoV-2 (day 14 post-vaccination)	Evaluation of an inactivated vaccine
	3–4 years (M/F)	SARS-CoV-2-2/human/CHN/CN1/2020						
		1 × 10^6^ TCID_50_ (IT) (day 22 post-vaccine)						
Gao et al. (2020) [41]	*n* = 4 control	Vaccinated with Al(OH)3 adjuvant (sham) or physiological saline (control) at 0, 7, and 14 days	NA (7)	Oropharynx, crissum, lung, rectum (3)	Severe interstitial pneumonia	CD3+, CD4+, CD8+, TNF-α, IFN-γ, IL-2, IL-4, IL-5, IL-6		
	3–4 years (M/F)	IM						
		SARS-CoV-2-2/human/CHN/CN1/2020						
		1 × 10^6^ TCID_50_ (IT) (22 days post-vaccine)						
Rhesus macaques	*n* = 6	SARS-CoV-2 nCoV-WA1-2020	Dyspnea (1/6) (7)	Nose, oropharynx, lung (1)	Minimal interstitial pneumonia subpleural spaces (3/6) (7) (7)	NA	NA	Testing of antiviral therapy
	Remdesivir			Low BAL titers (1)				
	(M/F)	2.6 × 10^6^ TCID_50_ (IT, IN, OC, PO)		No virus in BAL (3)	No extra pulmonary injury			
Williamson, B.N. et al. (2020) [42]	*n* = 6 control	Vehicle solution	Tachypnea, dyspnea	Nose, oropharynx, lung, and BAL (1)	Multifocal, mild to moderate, interstitial pneumonia (7)	NA	NA	
	(M/F)	SARS-CoV-2 CoV-WA1–2020			No extra pulmonary injury			
		2.6 × 10^6^ TCID_50_ (IT, IN, OC, PO)						
Rhesus macaques	*n* = 9	SARS-CoV-2 USA-WA1/2020	Reduced appetite (35)	Nose, pharynx, trachea, lung, gastrointestinal tract, liver, kidney, pneumocytes I & II, ciliated bronchial epithelial cells (1)	Acute interstitial pneumonia	Neutropenia	IgG anti- SARS-CoV-2 Spike protein (35)	Immune protection after a second exposure
					Consolidation	Lymphopenia (mild and transitory in high dose group)		
	6–12 years	initial inoculation			Edema	IFN-γ upregulation		
		1.1 × 10^6^, *n* = 3			Multiple Inflammatory foci			
	(M/F)	1.1 × 10^5^, *n* = 3			Hyaline membranes			
		1.1 × 10^4^ PFU, *n* = 3			Damage to type I and type II pneumocytes			
		(IN, IT)			Necrotic bronchiolar epithelium			
					Bronchiolar epithelial syncytial cells			
					No extra pulmonary injury			
Chandrashekar et al. (2020) [43]c	*n* = 9	SARS-CoV-2 USA-WA1/2020	No (14)	5 log_10_ reduction BAL & nasal viral loads (1)	NA	Increased virus-specific Nab titers		
		Second inoculation						
		day 35 post-initial infection						
	6–12 years	1.1 × 10^6^						
		1.1 × 10^5^						
	(M/F)	1.1 × 10^4^ PFU (IN, IT)						
Rhesus macaques	*n* = 7	SARS-CoV-2	Fever	Nose, oropharynx, lung, gut, spinal cord, bladder, rectum (3)	Thickened alveolar septa	Increase CD4+ T cells	IgG antibody anti-SARS-CoV-2 (14)	Immune protection after a second exposure
		WH-09/hum/2020	Weight loss		Macrophages accumulation in alveoli Degeneration alveolar epithelia			
	3–5 years		Posture change		Inflammatory infiltrates (5, 7)			
		Initial inoculation	Rapid breathing					
	(NA)	1 × 10^6^ TCID_50_ (IT)	Reduced appetite (28)					
Bao et al. (2020) [44]	(*n* = 4)	SARS-CoV-2	Transient temperature increase (14)	Negative	No pathology (5)	CD4+ T higher at 7 day post-exposure vs. post-initial exposure	Higher IgG antibody anti-SARS-CoV-2 (14) vs. initial exposure	
		WH-09/hum/2020						
	3–5 years							
	(NA)	second inoculation day 28 post-initial infection						
		1 × 10^6^ TCID_50_ (IT)						
Rhesus macaques	(*n* = 5)	SARS-CoV-2 WH-09/hum/2020	weight loss (IT route) (21)	Nasal, oropharynx, rectum (IG route)	Interstitial pneumonia (IT route)	NA	IgG anti-SARS-CoV-2 on 21 dpi (CJ route)	Viral infection routes
				Conjunctival (CJ route)	Mild interstitial pneumonia (CJ route)			
Deng, W. et al. (2020) [45]	3–5 years (M)	1 × 10^6^ TCID_50_ (IT, CJ, IG)		Lung, ileum, caecum (IT) (1)	No pneumonia (IG route) (7)			
Rhesus macaques	*n* = 4 young	SARS-CoV-2 CDC, Guangdong, China	Fever	Nose, oropharynx, trachea	Inflammatory cell infiltrates	Peak CD4+ T cells, CD8+ T cells, and monocytes (2)	IgG antibody anti-SARS-CoV-2 (4)	Pathogenesis of COVID-19 in different species of nonhuman primates
	*n* = 6 adult		Weight loss (21)	Bronchus, lung, rectum	Diffuse hemorrhage and necrosis			
	*n* = 4 old			Blood, spleen (2)	Swollen lymph nodes (hilar, mediastinal, mesenteric)			
		4.75 × 10^6^ PFU (IT, IN, CJ)			Pericardial effusion	Young stronger B cell responses vs. adults vs. old	IgG levels lower in young vs. adult vs. old	
	(NA)	(50% given to young)			Mild hepatic steatosis	Increased G-CSF, IL-1A, IL-8, IL-15, IL-18, MCP-1, MIP-1B, sCD40-L		
					splenic hemorrhage (4, 7, 12, 13, 15)			
Common Marmoset	*n* = 6	SARS-CoV-2	None	Nose, oropharynx, rectum	Broken pulmonary septum	NA	No	
		CDC, Guangdong		Blood (2)	Inflammatory infiltrates			
	Age = NA (M/F)	1 × 10^6^ PFU (IN)			Splenic hemorrhage			
					Swollen hepatocytes			
					Renal inflammatory infiltrate			
Cynomolgus macaques	*n* = 6	SARS-CoV-2 CDC, Guangdong	Fever	Nose, oropharynx, trachea	Inflammatory cell infiltrates	CD4+ T cells, CD8+ T cells, and monocytes (2)	IgG antibody anti-SARS-CoV-2 (4)	
			Weight loss	Bronchus, lung, rectum	Diffuse hemorrhage and necrosis			
	Adult	4.75 × 10^6^ PFU (IT, IN, PO)		Blood, spleen (2)	Swollen lymph nodes (hilar, mediastinal)	Young stronger B cell responses vs. adults vs. old		
					Hepatic steatosis			
Lu et al. (2020) [46]	(M/F)				Splenic hemorrhage	Increased G-CSF, IL-1A, IL-8, IL-15, IL-18, MCP-1, MIP-1B, sCD40-L		
Cynomolgus macaques	*n* = 4	SARS-Cov-2 BetaCoV/Munich/BavPat1/2020	serous nasal discharge (1/4 old monkey) (21)	Nose, oropharynx, lung	Foci pulmonary consolidation	NA	IgG antibody anti-SARS-CoV-2 (14)	Comparisons of pathogenesis between COVID-19, SARS-CoV and MERS-CoV
				Pneumocytes I & II	Diffuse alveolar damage			
	4–5 y (F)			Ciliated nasal, bronchial & bronchiolar epithelial cells	Hyaline membrane			
	15–20 years (F)	2 × 10^5^ TCID_50_ (IT, IN)			Multinucleated giant cells			
					Type I & II pneumocytes hyperplasia			
				Earlier detection in young (2) vs. old (4).	Alveolar edema			
					Leukocyte infiltration			
				Higher nasal replication in old vs. young	(4)			
Rockx et al. (2020) [48]	*n* = 10	MERS-CoV	No	Nose, oropharynx, lung	Foci pulmonary consolidation		IgG antibody anti-MERS-CoV (21)	
		EMC strain, accession no. NC_019843		Pneumocytes II	Alveolar edema			
	3–5 years	10^6^ TCID50		& rectal swabs (2)	Leukocyte infiltration			
					Type II pneumocytes hyperplasia			
	F	(IT, IN)						
	NA	NA	No	Nose, oropharynx, lung	Type I & II pneumocytes hyperplasia	NA	NA	
				Pneumocytes I & II	Alveolar edema (aged only)			
					Leukocyte infiltration			
					Hyaline membrane (aged only)			
Cynomolgus macaques	*n* = 6	2019-nCoV/USA-WA1-A12/2020	None (30)	Nose, eye, oropharynx, rectum (2)	CT scan: Ground glass appearance	Increased CXCL8, IL6, IL13, IL15, IL1RN, and TNF (6) in one macaque.	IgG antibody anti-SARS-CoV-2 spike S1 subunit (10)	Evaluation of medical interventions
					Reticulonodular opacities			
Finch et al. (2020) [47]	4–4.5 years (M/F)	3.65 × 10^6^ PFU (IT, IN)			Peri-bronchial thickening			
					Subpleural nodules			
					Alveolar dense consolidation (*n* = 1)			
					PET scan: FDG uptake lung and regional lymph nodes (2), mediastinal lymph nodes and spleen (6)			
African green monkey	*n* = 6	SARS-CoV-2-2/INMI1-/2020/Italy	Reduced appetite	Nasal, oropharynx, lung, rectum, pneumocytes I & II, alveolar macrophages (2)	Interstitial pneumonia	Increased CRP ¶ (*n* = 2)	IgG antibody against SARS-CoV-2 b (5)	Pathogenesis of COVID-19
					Bronchiolitis			
			Fever (31)					
Woolsey et al. (2020) [49]	NA	5 × 10^5^ PFU (IT, IN)			Edema	IL-8, IP-10, IL-12, IL-6, IFN-beta, IL10, and MCP-1 (2)		
					Hemorrhage			
					Hyaline membrane			
					Hyperplasia type II pneumocytes			
					Distention and flaccidity small intestines segments (5)			

*TCID_*50*_ Median Tissue Culture Infectious Dose at which 50% of the cells are infected, *PFU* plaque-forming unit, †*IT* intratracheal, *IN* intranasal, *CJ* intraconjunctival, *OC* ocular, *IG* intragastric, *PO* per oral. ‡ Viral replication: RNA copies (PCR), viral antigen (immunostaining), viral particles (electron microscopy). § *dpi* day post-inoculation, ¶ *CRP* C-reactive protein, || *NA* Not available. **Vaccine encoding spike protein variants: Full-length SARS-CoV-2 S protein, *S.dCT* Deletion of the cytoplasmic tail of SARS-CoV-2 S protein, *S.dTM* deletion of the transmembrane domain and cytoplasmic tail reflecting the soluble ectodomain, *S1* S1 domain with a fold on trimerization tag, *RBD* Receptor-binding domain with a fold on trimerization tag, *S.dTM.PP* a prefusion stabilized soluble ectodomain with deletion of the furin cleavage site, two proline mutations, and a fold on trimerization tag, *IM* Intramuscular

**Table 3:** Summary of studies using mice models of COVID-19

Species (ref)	Number age (gender)	Virus strain dose* (inoculation route)†	Clinical signs & observation duration (DPI) §	Viral replication‡ (DPI)	Pathology & sacrificing date (DPI)	Immune response	Seroconversion (DPI)	Outcome Measures
Mice	WT-BALB/c, *n* = 3	2 × 10^5^ TCID50 of P 4†† or 2 × 10^6^ of P 6†† (IN)	NA	Lung (3)	Mild lung pathology (2)	Mild inflammatory response	NA	Interferon response to SARS-CoV-2 infection
BALB/c:	SCID, *n* = 3		(14)	No difference in viral load WT vs. SCID	No difference in lung pathology WT vs. SCID (2, 4, 7, 14)			
WT¶								
SCID||	6–8 weeks (F)							
C57BL/6:	C57BL/6 *n* = 5	2 × 10^5^ TCID50 of P 4 or 2 × 10^6^ of P 6 (IN)	NA (14)	Lung (3)	Greater intra-alveolar hemorrhage and peribronchiolar inflammation in IFNar1−/− mice than WT and IL28r−/− mice (3)	Higher inflammatory response in IFNar1−/− vs. WT and IL28r−/− mice	NA	
WT	IFNar1−/−^¶¶^ *n* = 14							
Ifnar1−/−				Higher viral replication in IFNar1−/− mice vs. WT and IL28r−/− mice	(2, 4, 7, 14)			
Il28r−/−	6–8 weeks (F)							
Boudewijns et al. (2020) [56]	C57BL/6, *n* = 5	2 × 10^5^ TCID50 of P 4 or 2 × 10^6^ of P 6 (IN)	NA (14)	Lung (3)	Mild lung pathology (3)	Mild inflammatory response	NA	
	IL28r−/−, *n* = 5			No difference in viral load between WT and IL28r−/−	(2, 4, 7, 14)			
	6–8 weeks (F)							
Mice	hACE2 mice	SARS-CoV-2 (BetaCoV/Wuhan/IVDC-HB-01/2020|EPI_ISL_402119)	Slight	Highest viral load	Moderate interstitial pneumonia	MAC2, CD3+ T and CD19+ B cells in alveolar septum	IgG antibody response against SARS-CoV-2 (21)	Pathogenesis of COVID-19
hACE2‖‖ transgenic mice	(ACE2-HB-01)		Bristles	In lung (3)	Thickened alveolar septa			
			Weight loss	Intestine (1)				
	*n* = 19	10^5^ TCID_50_ (IN)	Arched back (14)	Alveolar macrophage, and alveolar epithelia (3)	Lymphocytes, macrophages, and monocytes infiltrates in the interstitial and alveolar space			
	6–11 months (M/F)							
					Bronchioles degeneration (3)			
					No pathology in intestine, spleen, heart, liver, kidney, brain, and testis			
					(1, 3, 5, 7)			
Bao et al. (2020) [50]	WT-HB-01 (*n* = 15)	SARS-CoV-2 (BetaCoV/Wuhan/IVDC-HB-01/2020|EPI_ISL_402119) 10^5^ TCID_50_ (IN)	No (14)	No viral RNA detectable in lung or intestine (1)	No (1, 3, 5, 7)	No	No	
	6–11 months (M/F)							
	Mock-treated hACE2 mice (*n* = 15)	PBS 50 μl (IN)	No (14)	No viral RNA detectable in lung or intestine	No (1, 3, 5, 7)	No	No	
	6–11 months (M/F)							
Mice	*n* = 5 6–8 weeks (M)	SARS-CoV-2 (BetaCoV/Hong Kong/VM20001061/2020 [KH1])	NA	NA	NA	NA	IgG antibody response against SARS-CoV and SARS-CoV-2 spike protein and RBD	Cross-reactivity of antibodies against SARS-CoV and SARS-CoV-2
	Infection	SARS-CoV (HK39849, SCoV)						
		10^5^ PFU (IN)						
BALB/c:	*n* = 5	Immunization with heat-inactivated plasma from SARS-CoV and SARS-CoV-2 (IP)	NA	NA	NA	NA	Cross-reactive antibody binding responses SARS-CoV-2 and SARS-CoV No cross-neutralization SARS-CoV-2 and SARS-CoV	
WT	6–8 weeks (M)							
Lv et al. (2020) [53]	Immunization							
	*n* = 6	Vehicle (IN)	NA	NA	NA	NA		
	6–8 weeks (M) control							
Transgenic mice	*n* = 7	SARS1/SARS2-RdRp §§	Improvement of pulmonary function	reduced lung viral load 10^2^ PFU/lobe (5)	Decreased lung hemorrhage (5) (5)	NA	NA	Antiviral therapy testing
C57BL/6^***^:	17 weeks (F)	10^3^ PFU (IN)	(5)					
Ces1c−/−		Remdesivir given at 1dpi						
Pruijssers et al. (2020) [55]	Remdesivir							
	*n* = 7	SARS1/SARS2-RdRp	Reduced pulmonary function by WPH††† (5)	Lung viral load	Lung hemorrhage (5) (5)	NA	NA	
	17 weeks (F)	10^3^ PFU (IN)		10^5^ PFU/lobe (5)				
	Control	Vehicle						
	*n* = 3	Mouse-adapted SARS-CoV-2 (BetaCoV/Wuhan/AMMS01/2020)	Weight loss old mice (5) (7)	Trachea, lung, heart, liver, and intestine, pneumocytes Type II	Thickened alveolar septa	Increased TNF-α, IL-1β, IL-6, and IL-5, MCP-1, G-CSF, and GM-CSF (3)	NA	Establishment of mouse-adapted SARSCoV-2 model of COVID19
	Young, 6 weeks (F)				Alveolar damage and focal exudation			
					Hemorrhage,			
	*n* = 3	7.2 × 10^5^ PFU (IN)		Viral replication similar in old vs. young cells (3)	Inflammatory cell infiltration	Higher and sustained cytokines levels in aged mice vs. young		
					Denaturation of endothelial tissues (3)			
Mice	Old, 9 months (F)				Lung pathology similar in old vs. young mice (3, 5, 7)			
BALB/c:								
WT	Control mice	NA	No weight loss	No viral protein	NO	NO		Evaluation of candidates vaccine
Gu et al. (2020) [54]	NA							
	*n* = 10	Immunization day 1, 14	NA	No viral replication detectable in lungs (5)	No	NA	Higher IgG antibody response against SARS-CoV-2 (14)	
	6–8 weeks	Challenged with mouse adapted SARS-CoV-2						
	Immunized with SARS-CoV-2 RBD-Fc protein	(IN), 4 weeks after second immunization						
	PBS control with aluminum adjuvant			High viral load in the trachea and lungs (5)	Focal perivascular and peribronchiolar inflammation Thickened alveolar septa	NA	NA	
Mice	*n* = 5	SARS-CoV-2	No (2)	No viral replication detectable in lung (2)	NA	NA	IgG1 ab1 protects hACE2 transgenic mice from SARS-CoV-2 infection. (2)	Evaluation of prophylaxis with monoclonal antibody
	6–9 weeks (F)	10^5^ PFU (IN)						
	C3B6: hACE2 mice							
	Immunization	Human monoclonal IgG1 antibody (12 h)						
C3B6:		Prior the virus challenges (IP)						
hACE2 transgenic mice	*n* = 6	SARS-CoV-2	No (2)	Viral replication 10^3^ PFU per lung (2)	NA	NA	No	
	6–9 weeks (F)	10^5^ PFU (IN)						
	C3B6: hACE2 mice							
BALB/c mice	Control	IgG1 m336 (no activity in vitro)						
Li et al. (2020) [51]	Balb/c, *n* = 5	Mouse ACE2 adapted SARS-CoV-2‡‡	No (2)	No viral replication detectable in lung lobe at different dosages (2)	NA	NA	IgG1 ab1 protected mice SARS-CoV-2 challenge (2)	
	10–12 months (F)							
		10^5^ PFU (IN)						
		Human monoclonal IgG1 ab1 antibody (12 h)						
		Prior the virus challenges (IP)						
Mice	hACE2 mice	SARS-CoV-2	Weight loss	Lung (2), brain (5)	NA (2, 5)	NA	NA	Evaluation of vaccine and therapy in mouse-adapted SARS-CoV-2 model
BALB/c: And hACE2	NA	10^5^ PFU (IN)	Mortality 40% (5) (5)					
Transgenic mice	BALB/c mice	SARS-CoV-2MA^§^	Pulmonary obstruction (WBP)^†††^	Upper airway		NA	NA	
Dinnon et al. (2020) [52]	*n* = 33	10^5^ PFU (IN)		Lung (2,4)	Greater lung inflammation and hemorrhage in old vs. young mice (2,4)			
	Young 12 weeks							
	BALB/c mice		Greater	Higher replication in old vs. young mice				
	*n* = 34		Weight and pulmonary					
	12 months		Function loss in old vs. young mice					
	Vaccination	SARS-CoV-2 spike (S) or nucleocapsid (N)	NA	Vaccine with spike S reduced lung and nasal turbinate titer (2)	NA	NA	NA	
	*n* = 8–10							
	10 weeks	Challenged 4 weeks post-inoculation with SARS-CoV-2 MA						
	BALB/c	10^5^ PFU (IN)						
	Prophylaxis	Subcutaneous administration interferon (IFN) lambda-1a 2 μg		Reduced SARS-CoV-2 MA replication in the lung (2)	NA	NA	NA	
	Therapy	18 h prior or 12 h after						
	BALB/c	SARS-CoV-2 MA						
	12 weeks	10^5^ PFU (IN)						
	*n* = NA							

*TCID_*50*_ Median Tissue Culture Infectious Dose at which 50% of the cells are infected, *PFU* plaque-forming unit, † *IN* intranasal, *IP* intraperitoneal. ‡ Viral replication: RNA copies (PCR), viral antigen (immunostaining), viral particles (electron microscopy). § *dpi* day post-inoculation. ¶ *WT* wild type, || *SCID* severe combined immunodeficiency (lacking functional T and B cells). ** SARS-CoV-2MA A recombinant mouse ACE2 adapted SARS-CoV-2 variant remodeled by introduction of two amino acid changes at the ACE2 binding pocket in the receptor-binding domain to facilitate efficient binding to mouse ACE2. †† P4 and P6: Number of serial passaging of patient SARS-CoV-2 on HuH7 and Vero-E6 cells. ‡‡ Remodeling of the SARS-CoV-2 spike protein in the receptor-binding domain to facilitate efficient binding to mouse ACE2. §§ Chimeric mouse-adapted SARS-CoV1 MA15 variant encoding the SARS-CoV2 RNA-dependent RNA polymerase (“SARS1/SARS2-RdRp”). ¶¶ Genetic ablation of type I (Ifnar1−/−), III interferon (IFN) receptors (Il28r−/−), and Signal transducer and activator of transcription 2 (STAT2−/−). ‖‖ *hACE2* chimera expressing human ACE2 receptor. *** C57BL/6 Mice Ces1c−/−: lack a serum esterase, an enzyme that is not present in humans, that reduces markedly the Remdesivir half-life. ††† *WPH* whole-body plethysmography

**Table 4:** Summary of studies using hamsters models of COVID-19

Species (ref)	Number age (gender)	Virus strain dose* (inoculation route)†	Clinical signs & observation duration (DPI) §	Viral replication‡ (DPI)	Pathology & sacrificing date (DPI)	Immune response	Seroconversion (DPI)	Outcome measures
Syrian hamsters Chan et al. (2020) [57]	*n* = 8	SARS-CoV-2 Hong Kong	Tachypnea	Nose, trachea, lung	Diffuse alveolar damage (exudative)	Upregulation of Interferon-γ and proinflammatory chemokine, cytokine genes expression	IgG antibody response against SARS-CoV-2 (7)	Viral transmission and immunoprophylaxis
			Weight loss	Intestine (high viral load¶) (2–7)				
	6–10 weeks (M/F) (Donor)	10^5^ PFU (IN)	Lethargy		Apoptosis			
			Ruffled furs	Blood (low viral load)	Diffuse alveolar damage (proliferative)	Early convalescent serum Immunoprophylaxis decreased nasal and lung viral load but not lung pathology or clinical signs		
			Hunched back posture (14)		Tissue repair			
					Intestinal villi damage and necrosis			
					Reduced spleen size (2–14) (2, 4, 7, 14)			
	n = 8	Direct contact with donor	Less weight loss than inoculated animals (14)	No difference in viral load inoculated animals vs. infected animals via contact (4)	No difference inoculated vs. infected by contact (2, 4, 7, 14)	NA	IgG antibody response against SARS-CoV-2 (7)	
	6–10 weeks (M/F)	Inoculated with 100 ul of PBS						
Syrian hamsters	*n* = 6 (mAb CC12.1 or CC12.23) **¶**	SARS-CoV-2 (USAWA1/2020)	Weight loss dose-dependent (5)	Reduced lung viral load	NA (5)	NA	Neutralizing antibody (5)	Immunoprophylaxis and therapy
		1X10^6^ PFU (IN)						
		12 h post-Ab infusion						
Rogers et al. (2020) [58]	*n* = 6 Control IgG1 (Den3) **‖**	SARS-CoV-2 (USAWA1/2020)	Weight loss (5)	No difference in lung viral loads control vs. low dose groups	NA (5)	NA	NA	
		1 × 10^6^ PFU (IN)						
		12 h post-Ab infusion						
Golden Syrian hamsters	(*n* = 9)) 4–5 weeks (M) (Donor)	SARS-CoV-2 BetaCoV/Hong Kong/VM20001061/2020	Weight loss (6)	Upper respiratory tract, nose, olfactory	Inflammatory infiltrates nasal turbinate Progressive lung consolidation (5 to 60%) Mononuclear cell infiltration.	CD3 positive T lymphocytes in peribronchial region (5)	IgG antibody response against SARS-CoV-2 (14)	Viral transmission
			Ruffled hair coat (5) (14)	Neurons, bronchus, lung	No extrapulmonary pathology			
				Kidney, duodenum				
		8 × 10^4^ TCID_50_ (IN)			No pathology in the intestine, spleen, heart, and brain (2, 5, 7) (2, 5, 7)			
Sia et al. (2020) [59]	(*n* = 9)	Infection via contact with donor hamster	Weight loss (6)	Detectable infectious viruses (9/9)	NA	NA	IgG antibody response against SARS-CoV-2 (14)	
	4–5 weeks (M)		Ruffled hair coat day (4) (14)	Day 1 post-contact				
	(Contact)			No difference in viral shedding contact vs. donor				
Syrian hamster	(*n* = 7)	SARS-CoV-2 (BetaCoV/Belgium/GHB03021/2020)	NA (4)	Lungs, blood, spleen, liver, upper & lower gastrointestinal tract	Multifocal necrotizing bronchiolitis,	Increased inflammation-related gene expression	NA	Host interferon response to SARS-CoV-2
	Age: NA (F)	2 × 10^5^ TCID_50_ (P4 virus) or 2 × 106 TCID_50_ (P6 virus) (IN)			Leukocyte infiltration			
	Wild type				Edema (4) (2, 3, 4)	No increase in serum levels of IL-6, IL-10, and IFN-γ (4)		
Hamster (STAT2−/− and IL28R-a −/−) strains	(*n* = 7)	Same as wild type	NA (4)	Greater levels of viral RNA in the lung, spleen, liver, blood, and upper and lower gastrointestinal tract in STAT2−/− hamster vs. WT and IL28ra−/−	Lung pathology and inflammation decreased in (STAT2−/−) but not in IL28R-a−/− hamsters (2,3,4) (2, 3, 4)	Increased IL-6 and IL-10 expression in lungs	NA	
	7–12 weeks (F)					No increase in serum levels of IL-6, IL-10, and IFN-γ (4)		
	STAT2−/−							
Boudewijns et al. (2020) [56]	(*n* = 7)	Same as wild type	NA (4)	Lungs, blood, spleen, liver, upper, & lower gastrointestinal tract	Bronchopneumonia and peribronchiolar inflammation (2,3,4) (2, 3, 4)	High (MMP)-9 levels in lung homogenates compare to WT	NA	
	5–7 weeks (F)							
				No differences in lung viral RNA levels in WT, vs. STAT2−/− vs. IL28R-a−/− hamsters		Increased IL-6 and IL-		
	IL28R-a−/−					10 expression in lungs		
						No increase in serum levels of IL-6, IL-10 and IFNγ (4)		

*TCID_*50*_ Median Tissue Culture Infectious Dose at which 50% of the cells are infected, *PFU* plaque-forming unit, † *IN* intranasal, ‡ viral replication: RNA copies (PCR), and or viral antigen (immunostaining), viral particles (electron microscopy). § *dpi* day post-inoculation, ¶ *mAb CC12.1* IP SARS-CoV-2-2-specific human neutralizing monoclonal antibodies, ‖ *IgG1 (Den3)* 2 mg of a dengue specific human IgG1

**Table 5:** Summary of studies using ferrets, cat, and dog models of SARS-CoV-2 infection

Species (ref)	Number age (gender)	Virus strain dose* (inoculation route)†	Clinical signs & observation duration (DPI)§	Viral replication‡ (DPI)	Pathology & sacrificing date (DPI)	Immune response	Seroconversion (DPI)	Outcome measures
Ferrets	*n* = 12	NMC-nCoV02/Korea	Increased body temperature	Nose, saliva, urine, and feces	Acute bronchiolitis	NA‡	IgG and serum-neutralizing antibody response against SARS-CoV-2 (12)	Pathogenesis of COVID-19
					Infiltrates of immune cells and debris (4)			
	12–20 months	10^5.5^ TCID_50_	Reduced activity `		(4, 8, 12)			
								Assess viral transmission
	M/F	IN	occasional coughs (12)	Trachea, lung, kidney, serum, and intestine (2)				
	*n* = 12	Control	NO (12)	NO	NO (4, 8, 12)	NO	NO	
	12–20 months							
	M/F							
Kim et al. (2020) [60]	*n* = 6 (naïve direct contact)	Direct contact	Increased body temperature (12)	All animals were infected (2)	(12)		IgG and serum-neutralizing antibody response against SARS-CoV-2 (12)	
	*n* = 6 (naïve indirect contact)	Indirect contacts	No increased body temperature (12)	Nose, feces (2 out of 6 animals) (4)	(12)		IgG antibody response against SARS-CoV-2 (12) Serum-neutralizing antibody response in 1 out of 6	
Ferrets Blanco-Melo et al. (2020) [61]	*n* = 6	USA-WA1/2020	NA (14)	Nose, trachea	NA (3, 14)	Reduced interferon type I and III response	NA	Host interferon response
	4 months	5 × 10^4^ PFU		(3,7)				
	Castrated male	IN				Increase proinflammatory chemokines and cytokines response		
Ferrets Richard et al. (2020) [62]	*n* = 4	BetaCoV/Munich/BavPat1/2020	NA (21)	Nose, throat, rectum	NA (21)	NA	IgG antibody response against SARS-CoV-2 (21)	Viral transmission
	6 months (F)	6.10^5^ TCID_50_						
	Donor	IN						
	*n* = 4	6 h post-inoculation co-housed with donor	NA (21)	Nose, throat, rectum (1)	NA (21)	NA	IgG antibody response against SARS-CoV-2 (21)	
	6 months (F)							
	direct contact							
	*n* = 4	1 dpi placed in an opposite cage (10 cm) of donor	NA (21)	Nose, throat, rectum (3)	NA (21)	NA	IgG antibody response against SARS-CoV-2 (21)	
	6 months (F)							
	Indirect contact							
Ferrets	*n* = 10	SARS-CoV-2 /F13/environment/2020/Wuhan	Fever	Nose, throat, and rectum (low titer) (4–8)	Severe lymphoplasmacytic, perivasculitis		IgG antibody response against SARS-CoV-2 (13)	Pathogenesis of COVID-19
	3–4 months F		Loss of appetite (20)		Vasculitis	NA		
		SARS-CoV2/CTan/human/2020/Wuhan]			Increased type II pneumocytes, macrophages, and neutrophils in the alveolar septa and alveolar lumen.			
		10^5^ PFU			Mild peribronchitis (13)			
		IN			(4, 20)			
	*n* = 8	SARS-CoV2/CTan/human/2020/Wuhan]	NA (14)	Nose, throat (2–8)	NA (2, 4, 8, 14)	NA	NA	
	3–4 months	10^5^ PFU						
	F	IT						
Cats	*n* = 10	SARS-CoV-2 /CTan/human/2020/Wuhan	NA (20)	Nose, soft palate, tonsil, trachea, lungs, and small intestines.	NA (3, 6, 10, 20)	NA	IgG antibody response against SARS-CoV-2 (10)	
	6–9 months	10^5^ PFU						
	M\F	IN						
	Sub-adult							
	*n* = 10	SARS-CoV-2 /CTan/human/2020/Wuhan	One cat died (3) (12)	Nose, soft palate, tonsil, trachea, lungs, and small intestines.	Extensive nasal, tonsil, tracheal, lung, and small intestine epithelial mucosal lesions (3) (3, 6, 11,12)	NA	IgG antibody response against SARS-CoV-2 (10)	
	70–100 days	10^5^ PFU						
	M/F	IN						
	Juvenile							
Dogs	*n* = 5	SARS-CoV-2-2/CTan/human/2020/Wuhan	NA	Rectum (2/5)	NA	NA	IgG antibody response against SARS-CoV-2 (14)	
	3-month beagles	10^5^ PFU						
		IN,						
Shi et al. (2020) [63]	*n* = 2	Exposed to donor	NA	No	NA	NA	No	
	3 month							
	Beagles							

*TCID_*50*_ Median Tissue Culture Infectious Dose at which 50% of the cells are infected, *PFU* plaque-forming unit, †*IT* intratracheal, *IN* intranasal, *CJ* intraconjunctival, *OC* ocular, *IG* intragastric, *PO* per oral, *IP* intraperitoneal, ‡ *viral replication* RNA copies (PCR), viral antigen (immunostaining), viral particles (electron microscopy), § *dpi* day post-inoculation

All the experimental animals were inoculated with SARS-CoV-2 with various strains, doses, and route of administration that differed across studies (Tables 2, 3, 4, and 5). Likewise, the time-point for tissue collection and pathological assessment were variables. These together precluded any comparisons between the animal models either intra-species or inter-species.

### Nonhuman primate models

#### Viral model

Rhesus macaques (*n* = 10) [37–46], cynomolgus (*n* = 3) [46–48], and African Green model (*n* = 1) [49] and common marmoset (*n* = 1) [46] were assessed as models for COVID-19 (Table 2). SARS-CoV-2 strains, dose, and route of inoculation were different across studies. Different doses of virus inoculum were compared in a single study and showed that viral load in the upper and lower respiratory tract, fever, weight loss, respiratory distress, and mortality were comparable regardless of the doses except for mild transient neutropenia and lymphopenia in the high dose group [43]. In contrast, the route of administration resulted in different pathological response as the intratracheal route elicited severe interstitial pneumonia, as compared with mild interstitial pneumonia and no pneumonia from the intraconjunctival and intragastric routes, respectively [45]. The animals were euthanized at different time-points post-inoculation ranging from 3 to 33 days.

#### Phenotype

The animals displayed variable clinical manifestations from none to fever, altered respiratory patterns, and other general signs (Table 2). The clinical manifestations were not different between old and young macaques [46–48]. Structural and ultrastructural examination of the respiratory tract were also variables including mild to moderate interstitial pneumonitis, edema, foci of diffuse alveolar damage with occasional hyaline membrane formation, and pneumocytes type II hyperplasia (Table 2). Old rhesus macaques exhibited more diffuse and severe interstitial pneumonia than young ones [47]. The extrapulmonary injury was investigated in five studies [40, 42, 43, 46, 49]. These revealed pathological changes in two studies [46, 49] including distention and flaccidity of the intestine, inflammatory cells infiltrating the jejunum, and colon, steatosis of the liver, and alteration of myocardial fiber architecture with increased mitochondrial density [46, 49]. No mortality was observed in any of the nonhuman primate models.

Comparisons between species of nonhuman primates were not possible except in one study, which suggested that rhesus macaques were superior to cynomolgus and common marmoset as models of human COVID-19 [46]. Other comparisons suggested that SARS-CoV elicited more severe lung pathology than SARS-CoV-2 and Middle East Respiratory Syndrome (MERS-CoV) [48] (Table 2).

#### Viral and host interaction

The virus replicated rapidly and at higher titers in the upper airway and lung in all four species [37–49]. The virus was detected in pneumocytes type I and II and ciliated epithelial cells of nasal, bronchial, and bronchiolar mucosa [37–49]. This differs from MERS-CoV where the virus was mainly present in type II pneumocytes [46] (Table 2). Replication of the virus was also demonstrated in jejunum, duodenum, colon, and rectum [37, 38, 40–49]. Viral genome was detected in the blood of rhesus macaques, cynomolgus, and marmoset in one study [46]. Viral replication of nasopharyngeal as well as anal swabs, and the lung in old macaques was higher than in young ones [47, 48].

SARS-CoV-2 infection-induced IgG antibodies response against the SARS-CoV-2 spike was noted in all species [37, 46, 48, 49] except in marmoset [46]. The antibodies were protective against a second exposure to the virus [43, 44]. There was no difference between males and females [37, 39–43, 46, 47]; however, young rhesus macaques had lower antibody titers than the old macaques [46]. The innate immune response to SARS-CoV-2 infection was variable with normal, high, or low leucocytes and lymphocyte counts [37, 46]. Occasional reduction of CD4^+^ and CD8^+^ T cell concentrations was documented [37] as well as the transitory release of various cytokines and chemokines at different days post-inoculation [37, 46, 49].

#### Drugs and vaccines

DNA and inactivated virus-based vaccines were evaluated and showed protection in these nonhuman primates. However, the DNA vaccine did not reduce the virus presence in the upper airway, while there was a residual small interstitial pneumonitis in the macaques that received the inactivated virus [40, 41]. This suggests that none of the virus tested so far displayed a comprehensive protection against SARS-CoV-2 infection. Several candidate DNA vaccines based in various forms of the SARS-CoV-2 Spike (S) protein were also tested in rhesus macaques [39]. The findings revealed that only the vaccine encoding the full-length (S) offered optimal protection against SARS-CoV-2 [64]. Nonhuman primates served also for the evaluation of antiviral therapies and medical interventions such as CT- and PET-scanners [47].

### Mouse models

#### Viral model

Wild type mice (BALB/c, C57BL/6), immunodeficient mice (SCID), chimeric mouse expressing human angiotensin-converting enzyme 2 (hACE2), and the RNA-dependent RNA polymerase (SARS1/SARS2-RdRp) were evaluated as models of COVID-19 (Table 3). Moreover, knockout (KO) mice were generated to test specific immunological pathways or therapy, including ablation of type I (IFNar1−/−), III interferon (IFN) receptors, (IL28r−/−), signal transducer and activator of transcription 2 (STAT2−/−), and serum esterase (Ces1c−/−).

Patient isolates of SARS-CoV-2 from different sources and variable times of passaging on various cell cultures or BALB/c mice were employed (Table 3). Mouse-adapted SARS-CoV-2 was developed using two methods. The first by serial passaging (up to 6) through the lungs of BALB/c mice until the virus spike receptor-binding domain (RBD) adapted to the murine ACE-2 [54]. In the second, using genetic engineering, the SARS-CoV-2 RBD was remodeled to enhance its binding efficiency to murine ACE2 [52].

#### Phenotype

The clinical signs and symptoms varied from none to mild weight loss, arched back, and slight bristles. Whole-body plethysmography was used to measure the respiratory function of the animals and showed a mild to moderate reduction in old more than in young (Table 3). Likewise, the pathological changes varied according to the experimental models and included peribronchiolar inflammation, lung edema, moderate multifocal interstitial pneumonia, lymphocyte infiltration, and intra-alveolar hemorrhage. Survival of hACE2 mice was decreased at 5-day post-inoculation and was attributed to high viral replication in the brain, while it was minimal in the lung, suggesting a different pathogenic mechanism of death from human COVID-19 [52]. Wild type mice showed no pathology as compared to hACE2 mice, indicating that the lack of human ACE2 receptor cannot be infected or inefficiently with SARS-CoV-2 [50, 56]. On the other hand, mouse-adapted SARS-CoV-2 exhibited more severe pathology, particularly in the aged mouse than hACE2 transgenic mouse, suggesting that these models may be more relevant for the study of human COVID-19 [52, 54]. However, whether the pathogenesis induced by the mouse-adapted SARS-CoV-2 is translatable to humans warrants further studies [52, 54].

#### Viral and host interaction

The virus replicated to high titers in the upper and lower respiratory tract in most of the genetically modified mice models but not in wild type. Viral replication was detected outside the respiratory tract in the intestine of hACE2 mice [50] as well as in the liver, and heart in mouse modified SARS-CoV-2 RBD [52]. Increased viral replication in KO mice IFNar1−/− suggested that interferon limited the viral replication [56].

Specific IgG antibodies against SARS-CoV-2 were documented in two studies (Table 3). The IgG antibodies were found to cross-react in their binding to the spike protein of SARS-CoV, however, with no cross-neutralization, hence suggesting the conservation of the same spike protein epitopes among coronaviruses [53]. Proinflammatory cytokines and chemokines were demonstrated in mouse-adapted SARS-CoV-2 and KO mouse (Table 3). The inflammatory response was significantly higher in the old than young mice.

#### Drugs and vaccines

Antiviral therapies, including remdesivir [55], IFN lambda [52], and human monoclonal IgG1 antibody against RBD [50], were tested in these mouse models and produced a protective effect. Likewise, vaccines using viral particles expressing SARS-2-S protein [52] or an RBD-based vaccine were tested and showed protection [55].

### Hamster

#### Viral model

Wild type Syrian hamsters and knockout hamsters for signal transducer and activator of transcription 2 (STAT2−/− lacking type I and III interferon signaling) and interleukin 28 receptors (IL28r−/− lacking IFN type III signaling) were reported as models for COVID-19. Patient isolate of SARS-CoV-2 from different sources and different passages on various cell cultures was used (Table 4). SARS-CoV-2 was administered intranasally at different titers to anesthetized hamsters. Viral transmission between hamsters was demonstrated either through direct contact or indirectly via airborne transmission.

#### Phenotype

The clinical manifestations included weight loss, which was consistently observed. Other clinical signs and symptoms such as rapid breathing, lethargy, ruffled furs, and hunched back posture were reported in one study [57]. The histopathological findings were variables according to the experimental models and ranged from lung consolidation to multifocal necrotizing bronchiolitis, leukocyte infiltration, and edema. STAT2−/− hamsters exhibited attenuated lung pathology as compared with IL28R-a−/− hamsters [56].

#### Viral and host interaction

The virus replicated to high titer in the upper and lower respiratory tract in most of the hamsters’ models. Viral replication was detected in the blood and kidney with a low concentration (Table 4). STAT2−/− hamsters had higher titers of infectious virus in the lung, viremia, and high levels of viral RNA in the spleen, liver, and upper and lower gastrointestinal tract in comparison with wild type and IL28R-a−/− hamsters. Specific IgG antibodies against SARS-CoV-2 were documented in the sera of hamsters at different time-points from virus inoculation ranging from 7 to 21 days. Increased expression of proinflammatory and chemokine genes was demonstrated in the lungs of the SARS-CoV-2 infected animals, however with no increase in circulating levels of proteins such as TNF, interferon-γ, and IL-6.

#### Drugs and vaccines

Immunoprophylaxis with early convalescent serum achieved a significant decrease in viral lung load but not in lung pathology [57].

### Ferrets, cat, and dog

#### Viral model and phenotype

Ferrets, cats, and dogs were administered intranasally or intratracheally with various doses and strains of SARS-CoV-2 (Table 5). Ferrets displayed elevated body temperature for several days associated with signs that differed according to the studies. These include decreased activity and appetite, sporadic cough, and no body weight loss [60–63]. No clinical signs were reported either in cats or in dogs.

#### Histopathological changes

Ferrets exhibited acute bronchiolitis [61, 63], with perivasculitis and vasculitis [63], but with no discernible pneumonia. Cats disclosed lesions in epithelial nasal, tracheal, and lung mucosa (Table 5).

#### Viral and host interaction

The virus replication and shedding were demonstrated in the upper airways and rectal swabs in ferrets and cats, but the extent to other tissues varied in ferrets from none to multiple organs, including the lung, blood, and urine. No viral RNA was detected in cats’ lungs. Dogs showed RNA-positive rectal swab but none in the upper or lower airways. Viral transmission between ferrets and cats was demonstrated either through direct contact [55] or indirectly via airborne route [62].

Ferrets, cats, and dogs exhibited specific antibody response against SARS-CoV-2 [60, 62, 63]. A study of the ferret immune response to SARS-CoV-2 revealed a subdued low interferon type I and type III response that contrasts with increased chemokines and proinflammatory cytokine IL6, which is reminiscent of the human response [61].

## Discussion

This systematic review of experimental animal models of SARS-CoV-2 induced- COVID-19 identified 13 peer-reviewed studies and 14 preprints that reported data on nonhuman primates [37–49], mice [50–56], hamsters [56–59], ferrets [60–63], cats, and dog [63] models of COVID-19. The main findings indicate that most of the animal models could mimic many features of mild human COVID-19 with a full recovery phenotype [3]. They also revealed that older animals display relatively more severe illness than the younger ones [38, 46, 48, 52, 54], which evokes human COVID-19 [3, 6]. However, none of the animal models replicated the severe or critical patterns associated with mortality as observed in humans with COVID-19 [3].

The results of this systematic review are consistent with studies of animal models of SARS-CoV and MERS-CoV, which failed to replicate the full spectrum of humans’ illness [65, 66]. Nonetheless, several features of mild COVID-19 in humans could be mirrored. High viral titers in the upper and lower respiratory tract and lung pathology were demonstrated in both large and small animal models. The pathology encompassed mild interstitial pneumonia, consolidation, and diffuse alveolar damage, albeit localized to a small lung area, edema, hyaline membrane formation, and inflammation. SARS-CoV-2 elicited specific antibody response against various viral proteins in the sera of most of the animal models.

This systematic review revealed that none of these newly established animal models replicated the common complications of human COVID-19 such as ARDS and coagulopathy [6, 8, 28–33, 67, 68]. ARDS can be particularly severe and results in refractory hypoxemia requiring maximum respiratory supportive measures in the intensive care unit [6, 67, 68]. The coagulopathy can lead to severe complications such as massive pulmonary embolism, cerebrovascular stroke, and mesenteric infarction, including in younger people [8, 28, 32, 33]. The pathology underlying these two complications were recently revealed by post-mortem studies disclosing diffuse alveolar damage involving the whole lung, hyaline membrane formation, and infiltration with inflammatory cells, thus leaving no air space open for ventilation [17, 18, 64, 69, 70]. It also detected the presence of diffuse and widespread thrombosis in the micro- and macro-circulation, including the pulmonary circulation compromising the lung perfusion [17, 18]. This double hit affecting the ventilation and perfusion simultaneously underlies the intractable hypoxemia that contributed to the high mortality. None of the animal models replicated the respiratory failure, thromboembolic manifestations, and their pathological expression, hence, indicating that a wide gap separates the animal models from the full spectrum of COVID-19 in humans.

The mechanisms of the lung injury and coagulopathy are not well understood, although several known pathways were postulated including cytokine storm leading to upregulation of tissue factor [5, 9, 24], activation/injury of the endothelium infected by the virus [30, 67, 71], complement activation [72], alveolar hypoxia promoting thrombosis [73], and autoantibodies against phospholipid and lupus anticoagulant [74, 75] modulating the hemostasis and coagulation cascade directly. Hence, the development of animal models that replicate the dysregulation of the inflammation and coagulation could be important, as these would allow the deciphering of the intimate mechanisms at play. This, in turn, may aid in identifying therapeutic targets and the testing of immunotherapy, anticoagulation, and thrombolytic interventions and thereby may improve the outcome.

Both antiviral and vaccine therapies were tested in rhesus macaques and mice infected with SARS-CoV-2 [40–42]. The antiviral drug stopped the viral replication and improved the pneumonitis [42, 55]. The vaccines induced an increase in titers of neutralizing antibodies in the sera that correlated with the decrease of viral replication and prevented the lung pathology [39–41]. These results represent a substantial proof of the concept of antiviral or vaccine efficacy against SARS-CoV-2 in animal models. However, because of the lack of overt clinical illness, the rapid clearance of the virus, and spontaneous improvement of the pneumonitis without lethality, the models do not permit the full assessment of the duration of the protection of the vaccines, or the effect of antiviral therapy on survival.

Since the emergence of SARS-CoV infection in 2003 [76], followed by the MERS-CoV in 2012 [77], and now with COVID-19, researchers have not been able to develop a model of coronavirus infection that reproduces the severity and lethality seen in humans [65, 66]. One of the well-known reasons lies in the difference of ACE-2 receptor binding domain structure across species [78]. Human and primates have conserved a comparable structure that allows binding with high affinity to the SARS-CoV-2 [78]. The hamsters, ferrets, and cats maintained an intermediate affinity, while mice exhibit very low affinity [78]. The latter explains why wild-type mouse does not support SARS-CoV-2 replication, and hence, the necessity to create a chimera that expresses human ACE-2, to enable the use of this species as a model of COVID-19 [50]. More recently, a study applying single-cell RNA sequencing to nonhuman primate uncovered another explanation that may underlie the difference between nonhuman primates and humans in expressing the complex phenotype of COVID-19 [79]. The study reveals that the cellular expression and distribution of ACE2 and TMPRSS2 which are essential for virus entry in the cells and its spread inside the body differ in the lung, liver, and kidney between the two species. ACE2 expression was found lower in pneumocytes type II and higher in ciliated cells in nonhuman primate lung as compared to humans [40]. This is particularly significant as type II pneumocytes are critical targets of SARS-CoV-2 in humans and the pathogenesis of lung injury/damage. Finally, the innate immune response including the defense system against viruses diverged during evolution both at the transcriptional levels and cellular levels, which may also explain why the SARS-CoV-2 hardly progresses in these animals outside the respiratory system [80]. Taken together, these fundamental differences represent a real challenge to the successful development of an animal model that reproduces human COVID-19.

This systematic review has a few limitations. First, it is the high number of preprints included in this study that have not been peer-reviewed. Second, the animal models from the same species were difficult to compare across studies, as they used different viral strain, inoculum size, route of administration, and timing of tissue collection.

## Conclusion: failure to reproduce a severe form of human COVID-19

This systematic review revealed that animal models of COVID19 mimic mild human COVID-19, but not the severe form COVID-19 associated with mortality. It also disclosed the knowledge generated by these models of COVID-19 including viral dynamic and transmission, pathogenesis, and testing of therapy and vaccines. Likewise, the study underlines the distinct advantages and limitations of each model, which should be considered when designing studies, interpreting pathogenic mechanisms, or extrapolating therapy or vaccines results to humans. Finally, harmonization of animal research protocols to generate results that are consistent, reproducible, and comparable across studies is needed.

## Supplementary information

**Additional file 1.** Data extraction, appraisal, and outcome.

**Additional file 2.** List of excluded studies.

## Data Availability

All data generated or analyzed during this study are included in this published article [and its supplementary information files].

## Abbreviations

COVID-19: Coronavirus disease 2019
SARS-CoV-2: Severe acute respiratory syndrome–coronavirus 2
ARDS: Acute respiratory distress syndrome
MOSD: Multiple organ dysfunction/failure
ACE: Angiotensin-converting enzyme
TMPRSS: Transmembrane serine protease
PRISMA: Preferred Reporting Items for Systematic Reviews and Meta-analysis
hACE2: Chimeric mouse expressing human angiotensin-converting enzyme 2
IFN: Interferon
STAT2: Signal transducer and activator of transcription 2
Ces1c: Serum esterase
RBD: Receptor-binding domain
MERS-CoV: Middle East Respiratory Syndrome
