# Supplementary material for: Evidence of a wide gap between COVID-19 in humans and animal models: a systematic review
Source: Crit Care. 2020 Oct 6;24:594. doi: 10.1186/s13054-020-03304-8 (PMC7537968; doi:10.1186/s13054-020-03304-8)
Supplement: Supplementary file 1 — Additional file 1. Data extraction, appraisal, and outcome. [file 13054_2020_3304_MOESM1_ESM.pdf]

Additional file 1. Data extraction, appraisal and outcome

We screened all the study titles and abstracts independently by two reviewers for relevance. We removed all duplicated references electronically and manually. We retrieved and evaluated full text when the abstract is uncertain about the significance of the study. Where there was disagreement on the inclusion of a full text, it was submitted to a third reviewer for further evaluation.

We accepted only original papers that 1) describe the clinical, pathology, virus replication, host response of animal models of COVID-19; and/or 2) evaluate prophylaxis, therapies, or vaccines, using animal models of COVID-19. We excluded all *in-vitro* and organoid-model studies, veterinary publications. Likewise, we omitted reviews, editorials, commentaries, and studies without full text.

Data extraction

We retrieved the full texts of the remaining papers that fulfilled the inclusion criteria restricted to the English language. The investigators were not blinded to the author's name, institution, or source of funding. Two (SE, MA, and BA) of the three reviewers extracted the data. Descriptive variables extracted were the population type (species, number of animals, age, and gender), study aim (biology, pathogenesis, testing of prophylaxis, drugs, or vaccine), the virus used (source of the strain, the titer of the inoculum, its route and timing of administration), clinical response (general, i.e., fever, weight loss, posture, piloerection, or organ-specific, i.e., breathing pattern, or alteration of mental status and outcome), pathology (structural, ultrastructural, immunohistochemistry of bronchial, pulmonary and extrapulmonary tissues), viral replication or detection (viral genome in cells and tissues, viral antigen or particles), and host response (i.e., antibodies response and target viral proteins, innate immunity, and coagulation), and the effects of prophylaxis, drugs, or vaccines (efficacy and safety). We documented the evidence that is agreed by the two reviewers in an excel spreadsheet for interpretation and analysis.

Outcome

Outcomes were organized according to species and categorized into phenotype (signs or symptoms; histopathology, time-course of the illness and outcome), viral (titer in each tissue organ, detection methods, duration of positivity), and host response (dynamic of seroconversion, inflammatory and hemostatic markers), therapy and vaccine (efficacy, and safety).
