# Supplementary material for: Evidence of a wide gap between COVID-19 in humans and animal models: a systematic review
Source: Crit Care. 2020 Oct 6;24:594. doi: 10.1186/s13054-020-03304-8 (PMC7537968; doi:10.1186/s13054-020-03304-8)
Supplement: Supplementary file 2 — Additional file 2. List of excluded studies. [file 13054_2020_3304_MOESM2_ESM.pdf]

Additional file 2. List of excluded studies

#	Excluded Studies JAN 1-MAY 20 2020	Exclusion reason
	**CATEGORY A**	**Unrelated to animal models**
1	Potential host range of multiple SARS-like coronaviruses and an improved ACE2-Fc variant that is potent against both SARS-CoV-2 and SARS-CoV-1	In vitro
2	Exceptional diversity and selection pressure on SARS-CoV and SARS-CoV-2 host receptor in bats compared to other mammals	Computational
3	Recombination and lineage-specific mutations led to the emergence of SARS-CoV-2	Computational
4	Genome based Evolutionary study of SARS-CoV-2 towards the Prediction of Epitope Based Chimeric Vaccine	Computational
5	Critical role of type III interferon in controlling SARS-CoV-2 infection, replication and spread in primary human intestinal epithelial cells	In vitro
6	TMPRSS2 and furin are both essential for proteolytic activation and spread of SARS-CoV-2 in human airway epithelial cells and provide promising drug targets	In vitro
7	Search for SARS-CoV-2 inhibitors in currently approved drugs to tackle COVID-19 pandemia	In vitro
8	SARS-coronavirus-2 replication in Vero E6 cells: replication kinetics, rapid adaptation and cytopathology	In vitro
9	Coronavirus and PARP expression dysregulate the NAD Metabolome: a potentially actionable component of innate immunity	In vitro
10	TMPRSS2 and TMPRSS4 mediate SARS-CoV-2 infection of human small intestinal enterocytes	In vitro
11	Positive selection of ORF3a and ORF8 genes drives the evolution of SARS-CoV-2 during the 2020 COVID-19 pandemic	Computational
12	Structural and functional analysis of a potent sarbecovirus neutralizing antibody	In vitro
13	Recapitulation of SARS-CoV-2 Infection and Cholangiocyte Damage with Human Liver Organoids	In vitro
14	A data-driven drug repositioning framework discovered a potential therapeutic agent targeting COVID-19	In silico
15	Human iPSC-Derived Cardiomyocytes are Susceptible to SARS-CoV-2 Infection	In vitro
16	Humanized Single Domain Antibodies Neutralize SARS-CoV-2 by Targeting Spike Receptor Binding Domain	In vitro
17	Multivariate Analyses of Codon Usage of SARS-CoV-2 and other betacoronaviruses	Computational
18	Spike protein binding prediction with neutralizing antibodies of SARS-CoV-2	Computational

19	A human monoclonal antibody blocking SARS-CoV-2 infection	In vitro
20	Fully human single-domain antibodies against SARS-CoV-2	Computational
21	The severe acute respiratory syndrome coronavirus 2 (SARS-CoV-2) envelope (E) protein harbors a conserved BH3-like sequence	Computational
22	Functional pangenome analysis suggests inhibition of the protein E as a readily available therapy for COVID-2019	Computational
23	Isolation and characterization of SARS-CoV-2 from the first US COVID-19 patient	In vitro
24	Analysis of the mutation dynamics of SARS-CoV-2 reveals the spread history and emergence of RBD mutant with lower ACE2 binding affinity	Computational
25	Structure-Based Design, Synthesis and Biological Evaluation of Peptidomimetic Aldehydes as a Novel Series of Antiviral Drug Candidates Targeting the SARS-CoV-2 Main Protease	In vitro
26	Artificial intelligence predicts the immunogenic landscape of SARS-CoV-2: toward universal blueprints for vaccine designs	Computational
27	Development of CRISPR as a prophylactic strategy to combat novel coronavirus and influenza	In vitro
28	Discovery of baicalin and baicalein as novel, natural product inhibitors of SARS-CoV-2 3CL protease in vitro	In vitro
29	Atazanavir inhibits SARS-CoV-2 replication and pro-inflammatory cytokine production	In vitro
30	Identification of antiviral drug candidates against SARS-CoV-2 from FDA-approved drugs	In vitro
31	In-silico analysis of SARS-CoV-2 genomes: Insights from SARS encoded non-coding RNAs	In silico
32	Rapid SARS-CoV-2 whole genome sequencing for informed public health decision making in the Netherlands	Human
33	BioLaboro: A bioinformatics system for detecting molecular assay signature erosion and designing new assays in response to emerging and reemerging pathogens	In silico
34	Characterisation of the transcriptome and proteome of SARS-CoV-2 using direct RNA sequencing and tandem mass spectrometry reveals evidence for a cell passage induced in-frame deletion in the spike glycoprotein that removes the furin-like cleavage site	In vitro
35	Crystal structure of SARS-CoV-2 nucleocapsid protein RNA binding domain reveals potential unique drug targeting sites	In vitro
36	Limited SARS-CoV-2 diversity within hosts and following passage in cell culture	In vitro
37	Exploring the genomic and proteomic variations of SARS-CoV-2 spike glycoprotein: a computational biology approach	Computational
38	Evidence of the Recombinant Origin and Ongoing Mutations in Severe Acute Respiratory Syndrome Coronavirus 2 (SARS-CoV-2)	Computational
39	A Library of Nucleotide Analogues Terminate RNA Synthesis Catalyzed by Polymerases of Coronaviruses Causing SARS and	In vitro

	COVID-19	
40	Virus-host interactome and proteomic survey of PMBCs from COVID-19 patients reveal potential virulence factors influencing SARS-CoV-2 pathogenesis	Human
41	Novel Immunoglobulin Domain Proteins Provide Insights into Evolution and Pathogenesis Mechanisms of SARS-Related Coronaviruses	Computational
42	RNA-GPS Predicts SARS-CoV-2 RNA Localization to Host Mitochondria and Nucleolus	Computational<br>Machine learning
43	Comparative genomic analysis revealed specific mutation pattern between human coronavirus SARS-CoV-2 and Bat-SARSr-CoV RaTG13	Computational<br>Genomic analysis
44	The IMPDH inhibitor merimepodib suppresses SARS-CoV-2 replication in vitro	In vitro
45	Preliminary identification of potential vaccine targets for the COVID-19 coronavirus (SARS-CoV-2) based on SARS-CoV immunological studies	Computational
46	Scutellaria baicalensis extract and baicalein inhibit replication of SARS-CoV-2 and its 3C-like protease in vitro	In vitro
47	Glycosaminoglycan binding motif at S1/S2 proteolytic cleavage site on spike glycoprotein may facilitate novel coronavirus (SARS-CoV-2) host cell entry	In vitro
48	Molecular Docking Analysis of Some Phytochemicals on Two Sars-Cov-2 Targets	Computational
49	Identification of potential treatments for COVID-19 through artificial intelligence-enabled phenomic analysis of human cells infected with SARS-CoV-2	In vitro
50	Genomic determinants of pathogenicity in SARS-CoV-2 and other human coronaviruses	Computational<br>Machine learning
51	Emergence of SARS-CoV-2 through Recombination and Strong Purifying Selection	Computational
52	A SARS-CoV-2-Human Protein-Protein Interaction Map Reveals Drug Targets and Potential Drug-Repurposing	In vitro
53	Computational Prediction of the Comprehensive SARS-CoV-2 vs. Human Interactome to Guide the Design of Therapeutics	Computational
54	HTCC as a highly effective polymeric inhibitor of SARS-CoV-2 and MERS-CoV	In vitro
55	The potential genetic network of human brain SARS-CoV-2 infection	Computational
56	Leveraging Deep Learning to Simulate Coronavirus Spike proteins has the potential to predict future Zoonotic sequences	Computational
57	Energetics based epitope screening in SARS CoV-2 (COVID 19) spike glycoprotein by Immuno-informatic analysis aiming to a suitable vaccine development	Computational
58	SARS-CoV-2 Isolation and Propagation from Turkish COVID-19 patients	Human
59	Emergence of RBD mutations in circulating SARS-CoV-2 strains	Computational

	enhancing the structural stability and human ACE2 receptor affinity of the spike protein	
60	Triphosphates of the Two Components in DESCOVY and TRUVADA are Inhibitors of the SARS-CoV-2 Polymerase	In vitro
61	Discovery of a 382-nt deletion during the early evolution of SARS-CoV-2	Human
62	The anti-HIV Drug Nelfinavir Mesylate (Viracept) is a Potent Inhibitor of Cell Fusion Caused by the SARS-CoV-2 Spike (S) Glycoprotein Warranting further Evaluation as an Antiviral against COVID-19 infections	In vitro
63	Characterization and treatment of SARS-CoV-2 in nasal and bronchial human airway epithelia	Human
64	Topoisomerase III-ß is required for efficient replication of positive-sense RNA viruses	In vitro
65	Potent human neutralizing antibodies elicited by SARS-CoV-2 infection	Human
66	Identification of a common deletion in the spike protein of SARS-CoV-2	Human
67	Genetic Variability of Human Angiotensin-Converting Enzyme 2 (hACE2) Among Various Ethnic Populations	Computational
68	Evolutionary origins of the SARS-CoV-2 sarbecovirus lineage responsible for the COVID-19 pandemic	Computational
69	Cytotoxicity evaluation of chloroquine and hydroxychloroquine in multiple cell lines and tissues by dynamic imaging system and PBPK model	In vitro
70	Design an efficient multi-epitope peptide vaccine candidate against SARS-CoV-2: An in silico analysis	In silico
71	Replication of SARS-CoV-2 in human respiratory epithelium	In vitro
72	Insights into The Codon Usage Bias of 13 Severe Acute Respiratory Syndrome Coronavirus 2 (SARS-CoV-2) Isolates from Different Geo-locations	Computational
73	Molecular Modeling Evaluation of the Binding Effect of Ritonavir, Lopinavir and Darunavir to Severe Acute Respiratory Syndrome Coronavirus 2 Proteases	Computational
74	On the interactions of the receptor-binding domain of SARS-CoV-1 and SARS-CoV-2 spike proteins with monoclonal antibodies and the receptor ACE2	Computational
75	CoV2ID: Detection and Therapeutics Oligo Database for SARS-CoV-2	Computational
76	Designing a multi-epitope peptide-based vaccine against SARS-CoV-2	In silico
77	The protein expression profile of ACE2 in human tissues	Human
78	Identification of potential vaccine candidates against SARS-CoV-2, A step forward to fight novel coronavirus 2019-nCoV: A Reverse Vaccinology Approach	In silico
79	Rapid metagenomic characterization of a case of imported COVID-19 in Cambodia	Human

80	The coronavirus proofreading exoribonuclease mediates extensive viral recombination	In vitro
81	Computational analysis of microRNA-mediated interactions in SARS-CoV-2 infection	Computational
82	Programmable low-cost DNA-based platform for viral RNA detection	In vitro
83	The FDA- approved gold drug Auranofin inhibits novel coronavirus (SARS-COV-2) replication and attenuates inflammation in human cells	In vitro
84	Replication of Equine arteritis virus is efficiently suppressed by purine and pyrimidine biosynthesis inhibitors	In vitro
85	Structure of replicating SARS-CoV-2 polymerase	In vitro
86	Interpretable detection of novel human viruses from genome sequencing data	Computational
87	Crystal structure of Nsp15 endoribonuclease NendoU from SARS-CoV-2	In vitro
88	Viral Architecture of SARS-CoV-2 with Post-Fusion Spike Revealed by Cryo-EM	Human
89	Epitope-based chimeric peptide vaccine design against S, M and E proteins of SARS-CoV-2 etiologic agent of global pandemic COVID-19: an in silico approach	Computational
90	Identification of variable sites in Sars-CoV-2 and their abundance profiles in time	Computational
91	Repurposing Pyramax® for the Treatment of Ebola Virus Disease: Additivity of the Lysosomotropic Pyronaridine and Non-Lysosomotropic Artesunate	In vitro
92	Inhibition of PIKfyve kinase prevents infection by EBOV and SARS-CoV-2	In vitro
93	Design of multi epitope-based peptide vaccine against E protein of human COVID-19: An immunoinformatics approach	Computational
94	In-depth Bioinformatic Analyses of Human SARS-CoV-2, SARS-CoV, MERS-CoV, and Other Nidovirales Suggest Important Roles of Noncanonical Nucleic Acid Structures in Their Lifecycles	Computational
95	Cryo-electron microscopy structure of the SADS-CoV spike glycoprotein provides insights into an evolution of unique coronavirus spike proteins	In vitro
96	Ultra-Low-Cost Integrated Silicon-based Transducer for On-Site, Genetic Detection of Pathogens	In vitro
97	Partial RdRp sequences offer a robust method for Coronavirus subgenus classification	Computational
98	Codon usage and evolutionary rates of the 2019-nCoV genes	Computational
99	The Essential Facts of Wuhan Novel Coronavirus Outbreak in China and Epitope-based Vaccine Designing against COVID-19	Computational
100	A molecular cell atlas of the human lung from single cell RNA sequencing	Human
101	Potential T-cell and B-cell Epitopes of 2019-nCoV	Computational
102	Azithromycin and ciprofloxacin have a chloroquine-like effect	In vitro

	on respiratory epithelial cells	
103	Coronavirus hemagglutinin-esterase and spike proteins co-evolve for functional balance and optimal virion avidity	In vitro
104	Characterising the epidemic spread of Influenza A/H3N2 within a city through phylogenetics	Computational
105	Bats Possess Unique Variants of the Antiviral Restriction Factor Tetherin	In vitro
106	In silico approach for designing of a multi-epitope based vaccine against novel Coronavirus (SARS-COV-2)	In silico
107	A facile method of mapping neutralizing HIV-1 epitopes	In vitro
108	Mutations, Recombination and Insertion in the Evolution of 2019-nCoV	Human
109	Cryo-EM structure of coronavirus-HKU1 haemagglutinin esterase reveals architectural changes arising from prolonged circulation in humans	In vitro
110	Single-cell analysis of upper airway cells reveals host-viral dynamics in influenza infected adults	Human
111	X-ray Structure of Main Protease of the Novel Coronavirus SARS-CoV-2 Enables Design of α-Ketoamide Inhibitors	Analytical
112	A unifying structural and functional model of the coronavirus replication organelle: tracking down RNA synthesis	In vitro
113	Inactivating porcine coronavirus before nuclei acid isolation with the temperature higher than 56 °C damages its genome integrity seriously	In vitro
114	Kallikrein 13: a new player in coronaviral infections	In vitro
115	A blueprint for the implementation of a validated approach for the detection of SARS-Cov2 in clinical samples in academic facilities	In vitro
116	Comparative ACE2 variation and primate COVID-19 risk	Computational
117	Broad and differential animal ACE2 receptor usage by SARS-CoV-2	Computational
118	Human organs-on-chips as tools for repurposing approved drugs as potential influenza and COVID19 therapeutics in viral pandemics	In vitro
119	Structural basis to design multi-epitope vaccines against Novel Coronavirus 19 (COVID19) infection, the ongoing pandemic emergency: an in silico approach	In silico
120	Molecular Detection of SARS-CoV-2 in Formalin Fixed Paraffin Embedded Specimens	In vitro
121	Computational simulations reveal the binding dynamics between human ACE2 and the receptor binding domain of SARS-CoV-2 spike protein	Computational
122	Atlas of ACE2 gene expression in mammals reveals novel insights in transmisson of SARS-Cov-2	Computational
123	Integrated analyses of single-cell atlases reveal age, gender, and smoking status associations with cell type-specific expression of mediators of SARS-CoV-2 viral entry and highlights	Computational

	inflammatory programs in putative target cells	
124	Scrutinizing the SARS-CoV-2 protein information for the designing an effective vaccine encompassing both the T-cell and B-cell epitopes	Computational
125	SARS-CoV-2 is sensitive to type I interferon pretreatment	In vitro
126	SARS-CoV-2 and SARS-CoV Spike-RBD Structure and Receptor Binding Comparison and Potential Implications on Neutralizing Antibody and Vaccine Development	In vitro
127	Broad-spectrum antiviral activity of naproxen: from Influenza A to SARS-CoV-2 Coronavirus	In vitro
128	Association of Blood Glucose Control and Outcomes in Patients with COVID-19 and Pre-existing Type 2 Diabetes.	Human-retrospective
129	Analysis of the susceptibility of lung cancer patients to SARS-CoV-2 infection.	Human
130	Surge capacity of intensive care units in case of acute increase in demand caused by COVID-19 in Australia.	Human-survey
131	Association of Inpatient Use of Angiotensin Converting Enzyme Inhibitors and Angiotensin II Receptor Blockers with Mortality Among Patients With Hypertension Hospitalized With COVID-19	Human-retrospective
132	[The epidemiological characteristics of an outbreak of 2019 novel coronavirus diseases (COVID-19) in China].	Human cohort Chinese language
133	Expression of SARS-CoV-2 Entry Molecules ACE2 and TMPRSS2 in the Gut of Patients With IBD	Human
134	Attenuated SARS-CoV-2 variants with deletions at the S1/S2 junction	In vitro
135	SARS-CoV-2: Structural Diversity, Phylogeny, and Potential Animal Host Identification of Spike Glycoprotein	Computational
136	EZH2-mediated H3K27me3 inhibits ACE2 expression	In vitro
137	TMPRSS2 and TMPRSS4 promote SARS-CoV-2 infection of human small intestinal enterocytes	Human
138	SARS-CoV-2 Receptor ACE2 Is an Interferon-Stimulated Gene in Human Airway Epithelial Cells and Is Detected in Specific Cell Subsets across Tissues	Human
139	SARS-CoV2 (COVID-19) Structural/Evolution Dynamicome: Insights into functional evolution and human genomics.	Computational
140	On spatial molecular arrangements of SARS-CoV2 genomes of Indian patients	Computational
141	Prediction analysis of SARS-COV-2 entry in Livestock and Wild animals	In silico
142	Functional and Genetic Analysis of Viral Receptor ACE2 Orthologs Reveals a Broad Potential Host Range of SARS-CoV-2	In vitro
143	ACE2-Variants Indicate Potential SARS-CoV-2-Susceptibility in Animals: An Extensive Molecular Dynamics Study	In vitro
144	Global view on virus infection in non-human primates and implication for public health and wildlife conservation	Computational
145	SARS-CoV-2 spike protein predicted to form stable complexes with host receptor protein orthologues from mammals	Computational

146	An artificial intelligence system reveals liquiritin inhibits SARS-CoV-2 by mimicking type I interferon	Computational
147	Comparison of SARS-CoV-2 spike protein binding to human, pet, farm animals, and putative intermediate hosts ACE2 and ACE2 receptors	In vitro
148	IgA MAb blocks SARS-CoV-2 Spike-ACE2 interaction providing mucosal immunity	In vitro
149	Selectomic and Evolvability Analyses of the Highly Pathogenic Betacoronaviruses SARS-CoV-2, SARS-CoV, and MERS-CoV	Computational
150	SARS-CoV-2 ORF3b is a potent interferon antagonist whose activity is further increased by a naturally occurring elongation variant	In vitro
151	Multiple expression assessments of ACE2 and TMPRSS2 SARS-CoV-2 entry molecules in the urinary tract and their associations with clinical manifestations of COVID-19	Computational
152	Boceprevir, GC-376, and calpain inhibitors II, XII inhibit SARS-CoV-2 viral replication by targeting the viral main protease	In vitro
153	Rapid isolation and profiling of a diverse panel of human monoclonal antibodies targeting the SARS-CoV-2 spike protein	In vitro
154	The SARS-CoV-2 conserved macrodomain is a highly efficient ADP-ribosylhydrolase enzyme	In vitro
155	Identification of Drugs Blocking SARS-CoV-2 Infection using Human Pluripotent Stem Cell-derived Colonic Organoids	In vitro
156	Pathogen Reduction of SARS-CoV-2 Virus in Plasma and Whole Blood using Riboflavin and UV Light	In vitro
157	Shotgun Transcriptome and Isothermal Profiling of SARS-CoV-2 Infection Reveals Unique Host Responses, Viral Diversification, and Drug Interactions	In vitro
158	Feline coronavirus drug inhibits the main protease of SARS-CoV-2 and blocks virus replication	In vitro
159	Epigenetic regulator miRNA pattern differences among SARS-CoV, SARS-CoV-2 and SARS-CoV-2 world-wide isolates delineated the mystery behind the epic pathogenicity and distinct clinical characteristics of pandemic COVID-19	In silico
160	Tracing two causative SNPs reveals SARS-CoV-2 transmission in North America population	Computational
161	Lung biopsy cells transcriptional landscape from COVID-19 patient stratified lung injury in SARS-CoV-2 infection through impaired pulmonary surfactant metabolism	Human
162	Comparative analysis of antiviral efficacy of FDA-approved drugs against SARS-CoV-2 in human lung cells: Nafamostat is the most potent antiviral drug candidate	In vitro
163	Traffic-derived particulate matter and angiotensin-converting enzyme 2 expression in human airway epithelial cells	In vitro
164	Bulk and single-cell gene expression profiling of SARS-CoV-2 infected human cell lines identifies molecular targets for therapeutic intervention	In vitro

165	Potential antiviral options against SARS-CoV-2 infection	Human
166	Insights into molecular evolution recombination of pandemic SARS-CoV-2 using Saudi Arabian sequences	In vitro
167	Androgen Regulates SARS-CoV-2 Receptor Levels and Is Associated with Severe COVID-19 Symptoms in Men	In vitro
168	In Vitro Inhibition of SARS-CoV-2 Infection by Bovine Lactoferrin	In vitro
169	Neutralization of SARS-CoV-2 by destruction of the prefusion Spike	In vitro
170	Evaluation Of SYBR Green Real Time PCR For Detecting SARS-CoV-2 From Clinical Samples	Human
171	Transcriptional profiling reveals TRPM5-expressing cells involved in viral infection in the olfactory epithelium	In vitro
172	Low doses of radiation therapy increase the immunosuppressive profile of lung macrophages via IL-10 production and IFNγ/IL-6 suppression: a therapeutic strategy to counteract lung inflammation?	In vitro
173	Novel NGS Pipeline for Virus Discovery from a Wide Spectrum of Hosts and Sample Types	Human
174	Evolutionary arms race between virus and host drives genetic diversity in bat SARS related coronavirus spike genes	In vitro
175	The Host Cell ViroCheckpoint: Identification and Pharmacologic Targeting of Novel Mechanistic Determinants of Coronavirus-Mediated Hijacked Cell States	In vitro
176	[Exploring the mechanism of liver enzyme abnormalities in patients with novel coronavirus-infected pneumonia]	Human<br>Chinese language
177	Development and validation of a UHPLC-MS/MS method for quantification of the prodrug remdesivir and its metabolite GS-441524: a tool for clinical pharmacokinetics of SARS-CoV-2/COVID-19 and Ebola virus disease	In vitro
178	Immunoinformatic identification of B cell and T cell epitopes in the SARS-CoV-2 proteome	Computational
179	Biophysical characterization of the SARS-CoV-2 spike protein binding with the ACE2 receptor and implications for infectivity	Computational
180	The Red Queen's Crown: an evolutionary arms race between coronaviruses and mammalian species reflected in positive selection of the ACE2 receptor among many species	Computational
181	Infection Groups Differential (IGD) Score Reveals Infection Ability Difference between SARS-CoV-2 and Other Coronaviruses	Computational
182	Origin of Novel Coronavirus (COVID-19): A Computational Biology Study using Artificial Intelligence	Computational
183	Spike mutation pipeline reveals the emergence of a more transmissible form of SARS-CoV-2	in vitro
184	Intra-genome variability in the dinucleotide composition of SARS-CoV-2	Computational
185	Identification of five antiviral compounds from the Pandemic Response Box targeting SARS-CoV-2	Computational

186	SARS-CoV-2 is well adapted for humans. What does this mean for re-emergence?	Computational
187	Architecture and self-assembly of the SARS-CoV-2 nucleocapsid protein	In vitro
188	Distinct conformational states of SARS-CoV-2 spike protein	In vitro
189	SARS-CoV-2 amino acid substitutions widely spread in the human population are mainly located in highly conserved segments of the structural proteins	Computational
190	Broad sarbecovirus neutralizing antibodies define a key site of vulnerability on the SARS-CoV-2 spike protein	In vitro
191	Predicting the Immunogenicity of T cell epitopes: From HIV to SARS-CoV-2	Computational
192	An integrated in silico immuno-genetic analytical platform provides insights into COVID-19 serological and vaccine targets	Computational
193	COVID-19: Viral-host interactome analyzed by network based-approach model to study pathogenesis of SARS-CoV-2 infection	Computational
194	CoV-AbDab: the Coronavirus Antibody Database	Computational
195	In Silico Trial to test COVID-19 candidate vaccines: a case study with UISS platform	In Silico
196	Children's Hospital Los Angeles COVID-19 Analysis Research Database (CARD) – A Resource for Rapid SARS-CoV-2 Genome Identification Using Interactive Online Phylogenetic Tools	Computational
197	Identification of conserved epitopes in SARS-CoV-2 spike and nucleocapsid protein	Computational
198	SARS-CoV-2 Spike Glycoprotein Receptor Binding Domain is Subject to Negative Selection with Predicted Positive Selection Mutations	Computational
199	ACE2 interaction networks in COVID-19: a physiological framework for prediction of outcome in patients with cardiovascular risk factors	Computational
200	The UCSC SARS-CoV-2 Genome Browser	Computational
201	A Combined approach of MALDI-TOF Mass Spectrometry and multivariate analysis as a potential tool for the detection of SARS-CoV-2 virus in nasopharyngeal swabs	In vitro
202	Structural basis for translational shutdown and immune evasion by the Nsp1 protein of SARS-CoV-2	In vitro
203	A comprehensive germline variant and expression analyses of ACE2, TMPRSS2 and SARS-CoV-2 activator FURIN genes from the Middle East: Combating SARS-CoV-2 with precision medicine	Computational
204	Deep phylogenetic analysis of Orthocoronavirinae genomes traces the origin, evolution and transmission route of 2019 novel coronavirus	Computational
205	Epitope-Based Peptide Vaccine Against Severe Acute Respiratory Syndrome-Coronavirus-2 Nucleocapsid Protein: An in silico Approach	In silico
206	In silico design and validation of commercial kit GPS™ CoVID-19	In silico

	dtec-RT-qPCR Test under criteria of UNE/EN ISO 17025:2005 and ISO/IEC 15189:2012	
207	Comparative Genomic Analysis of Rapidly Evolving SARS-CoV-2 Viruses Reveal Mosaic Pattern of Phylogeographical Distribution	Computational
208	The SARS-CoV-2-like virus found in captive pangolins from Guangdong should be better sequenced	Computational
209	SARS-CoV-2 transcriptome analysis and molecular cataloguing of immunodominant epitopes for multi-epitope based vaccine design	In silico
210	A putative new SARS-CoV protein, 3a*, encoded in an ORF overlapping ORF3a	Computational
211	Crystal structures of SARS-CoV-2 ADP-ribose phosphatase (ADRP): from the apo form to ligand complexes	In vitro
212	Whole Genome Comparison of Pakistani Corona Virus with Chinese and US Strains along with its Predictive Severity of COVID-19	Computational
213	Knowledge, attitude and practice of secondary school students toward COVID-19 epidemic in Italy: a cross selectional study	Human-survey
214	Analysis of the SARS-CoV-2 spike protein glycan shield: implications for immune recognition	In vitro
215	Evolutionary Dynamics And Geographic Dispersal Of Beta Coronaviruses In African Bats	Computational
216	Potent programmable antiviral against dengue virus in primary human cells by Cas13b RNP with short spacer and delivery by virus-like particle	in silico
217	ACE2 polymorphisms and individual susceptibility to SARS-CoV-2 infection: insights from an in-silico study	in-silico
218	COVID-19 coronavirus vaccine design using reverse vaccinology and machine learning	Computational
219	A Large-scale Drug Repositioning Survey for SARS-CoV-2 Antivirals	In vitro
220	Regulation of angiotensin converting enzyme 2 (ACE2) in obesity: implications for COVID-19	Computational
221	Knowledge synthesis from 100 million biomedical documents augments the deep expression profiling of coronavirus receptors	Computational
222	Controlling the SARS-CoV-2 outbreak, insights from large scale whole genome sequences generated across the world	Computational
223	SARS-CoV-2 receptor and entry genes are expressed by sustentacular cells in the human olfactory neuroepithelium	Human
224	Significant expression of FURIN and ACE2 on oral epithelial cells may facilitate the efficiency of SARS-CoV-2 entry	Computational
225	When Darkness Becomes a Ray of Light in the Dark Times: Understanding the COVID-19 via the Comparative Analysis of the Dark Proteomes of SARS-CoV-2, Human SARS and Bat SARS-Like Coronaviruses	Computational
226	Re-analysis of SARS-CoV-2 infected host cell proteomics time-	Computational

	course data by impact pathway analysis and network analysis. A potential link with inflammatory response	
227	Comparison of commercial RT-PCR diagnostic kits for COVID-19	In vitro
228	The genomic variation landscape of globally-circulating clades of SARS-CoV-2 defines a genetic barcoding scheme	Computational
229	Comparative analyses of SAR-CoV2 genomes from different geographical locations and other coronavirus family genomes reveals unique features potentially consequential to host-virus interaction and pathogenesis	Computational
230	The global population of SARS-CoV-2 is composed of six major subtypes	Computational
231	SARS-CoV-2, an evolutionary perspective of interaction with human ACE2 reveals undiscovered amino acids necessary for complex stability	Computational
232	SARS-CoV-2 exhibits intra-host genomic plasticity and low-frequency polymorphic quasispecies	Computational
233	Master Regulator Analysis of the SARS-CoV-2/Human interactome	Computational
234	SARS-CoV-2 and ORF3a: Non-Synonymous Mutations and Polyproline Regions	Computational
235	SARS-CoV-2 might manipulate against its host the immunity RNAi/Dicer/Ago system	Computational
236	Pangolin homology associated with 2019-nCoV	Computational
237	IDseq – An Open Source Cloud-based Pipeline and Analysis Service for Metagenomic Pathogen Detection and Monitoring	Computational
238	The accelerated infectious disease risk in the Anthropocene: more outbreaks and wider global spread	Computational
239	Orthogonal genome-wide screenings in bat cells identify MTHFD1 as a target of broad antiviral therapy	Computational
240	Cross-scale dynamics and the evolutionary emergence of infectious diseases	Computational
241	Broad Host Range of SARS-CoV-2 Predicted by Comparative and Structural Analysis of ACE2 in Vertebrates	Computational
242	Modeling the epidemic dynamics and control of COVID-19 outbreak in China.	Computational
243	Genome Composition and Divergence of the Novel Coronavirus (2019-nCoV) Originating in China	Computational
244	Computers and viral diseases. Preliminary bioinformatics studies on the design of a synthetic vaccine and a preventative peptidomimetic antagonist against the SARS-CoV-2 (2019-nCoV, COVID-19) coronavirus	Computational
245	Receptor Recognition by the Novel Coronavirus from Wuhan: an Analysis Based on Decade-Long Structural Studies of SARS Coronavirus.	Computational
246	Establishment and validation of a pseudovirus neutralization assay for SARS-CoV-2	In vitro
247	Crystal structure of SARS-CoV-2 main protease provides a basis	In vitro

	for design of improved Î±-ketoamide inhibitors	
248	Inhibition of SARS-CoV-2 (Previously 2019-nCoV) Infection by a Highly Potent Pan-Coronavirus Fusion Inhibitor Targeting Its Spike Protein That Harbors a High Capacity to Mediate Membrane Fusion	In vitro
249	Spike protein recognition of mammalian ACE2 predicts the host range and an optimized ACE2 for SARS-CoV-2 infection	Computational
250	Aerosol and Surface Distribution of Severe Acute Respiratory Syndrome Coronavirus 2 in Hospital Wards, Wuhan, China, 2020	In vitro
251	Supporting pandemic response using genomics and bioinformatics: a case study on the emergent SARS-CoV-2 outbreak	In vitro
252	The SARS-CoV-2 receptor ACE2 expression of maternal-fetal interface and fetal organs by single-cell transcriptome study	Computational
253	Clinical features of the first cases and a cluster of Coronavirus Disease 2019 (COVID-19) in Bolivia imported from Italy and Spain	Human
254	Bioinformatic analysis indicates that SARS-CoV-2 is unrelated to known artificial coronaviruses	Computational
255	Rapid community-driven development of a SARS-CoV-2 tissue simulator	Computational
256	Identification and enrichment of SECReTE cis-acting RNA elements in the Coronaviridae and other (+) single-strand RNA viruses	Computational
257	LY6E Restricts the Entry of Human Coronaviruses, including the currently pandemic SARS-CoV-2	In vitro
258	eCovSens-Ultrasensitive Novel In-House Built Printed Circuit Board Based Electrochemical Device for Rapid Detection of nCovid-19 antigen, a spike protein domain 1 of SARS-CoV-2	In vitro
259	Coronavirus, as a source of pandemic pathogens.	Computational
260	A new phylogenetic protocol: Dealing with model misspecification and confirmation bias in molecular phylogenetics	Computational
261	Parallel global profiling of plant TOR dynamics reveals a conserved role for LARP1 in protein translation	In vitro
262	MicrobioLink: An integrated computational pipeline to infer functional effects of microbiome-host interactions	Computational
263	Omics BioAnalytics: Reproducible Research using R Shiny and Alexa	Computational
264	Microneedle array delivered recombinant coronavirus vaccines: Immunogenicity and rapid translational developmen	In vitro
265	Ecological processes underlying the emergence of novel enzootic cycles—arboviruses in the neotropics as a case study	Computational
266	High Throughput Designing and Mutational Mapping of RBD-ACE2 Interface Guide Non-Conventional Therapeutic Strategies for COVID-19	Computational

	CATEGORY B	Animal models without SARS-CoV-2 inoculation
1	A novel antiviral formulation inhibits a range of enveloped viruses	Other viruses
2	Structure-based design of hepatitis C virus E2 glycoprotein improves serum binding and cross-neutralization	No inoculation
3	Self-amplifying RNA SARS-CoV-2 lipid nanoparticle vaccine induces equivalent preclinical antibody titers and viral neutralization to recovered COVID-19 patients	S PROTEIN/VACCINE
4	Treating Influenza and SARS-CoV-2 via mRNA-encoded Cas13a	Influenza A virus
5	Structural Basis for Potent Neutralization of Betacoronaviruses by Single-domain Camelid Antibodies	NO INOCULATION
6	Tissue-resident CD8+ T cells drive age-associated chronic lung sequelae following viral pneumonia	Primary influenza virus
7	Predicting the presence and titer of rabies virus neutralizing antibodies from low-volume serum samples in low-containment facilities	Murine leukemia virus Not SARS-CoV-
8	Social Stress Alters Immune Response and Results in Higher Viral Load During Acute SIV Infection in a Pigtailed Macaque Model of HIV	HIV NOT SARS-CoV-2
9	Mucin 4 Protects Female Mice from Coronavirus Pathogenesis	Sars-Cov-1 NOT SARS-CoV-2
10	Nonmedical Masks in Public for Respiratory Pandemics: Droplet Retention by Two-Layer Textile Barrier Fully Protects Germ-free Mice from Bacteria in Droplets	NO INOCULATION
11	Safety, pharmacokinetics, and liver-stage Plasmodium cynomolgi effect of high-dose ivermectin and chloroquine in Rhesus Macaques	NO INOCULATION
12	Seasonal weight changes in laboratory ferrets	NO INOCULATION
13	An orally bioavailable broad-spectrum antiviral inhibits SARS-CoV-2 and multiple endemic, epidemic and bat coronavirus	MERS-CoV
14	Immunity-and-Matrix-Regulatory Cells Derived from Human Embryonic Stem Cells Safely and Effectively Treat Mouse Lung Injury and Fibrosis	NO INOCULATION
15	Cross-reactive neutralization of SARS-CoV-2 by serum antibodies from recovered SARS patients and immunized animals	NO INOCULATION
16	Remdesivir inhibits renal fibrosis in obstructed kidneys	NO INOCULATION
17	No evidence that androgen regulation of pulmonary TMPRSS2 explains sex-discordant COVID-19 outcomes	NO INOCULATION
18	Artesunate interacts with Vitamin D receptor to reverse mouse model of sepsis-induced immunosuppression via enhancing autophagy	Pseudomonas Aeruginosa
19	Fractal analysis of muscle activity patterns during locomotion: pitfalls and how to avoid them	No inoculation

20	An orally bioavailable broad-spectrum antiviral inhibits SARS-CoV-2 in human airway epithelial cell cultures and multiple coronaviruses in mice	MERS-CoV
21	Single-Dose, Intranasal Immunization with Recombinant Parainfluenza Virus 5 Expressing Middle East Respiratory Syndrome Coronavirus (MERS-CoV) Spike Protein Protects Mice from Fatal MERS-CoV Infection.	MERS-CoV
22	Key residues of the receptor binding motif in the spike protein of SARS-CoV-2 that interact with ACE2 and neutralizing antibodies	No inoculation
23	Acute chloroquine poisoning: A comprehensive experimental toxicology assessment of the role of diazepam	Drug testing
24	An effective CTL peptide vaccine for Ebola Zaire Based on Survivors' CD8+ targeting of a particular nucleocapsid protein epitope with potential implications for COVID-19 vaccine design	Ebola
25	Analysis and annotation of genome-wide DNA methylation patterns in two nonhuman primate species using the Infinium Human Methylation 450K and EPIC BeadChips	No inoculation
26	Mesenchymal stem cells with overexpression of Angiotensin-converting enzyme-2 improved the microenvironment and cardiac function in a rat model of myocardial infarction	No inoculation
27	Background mechanisms of olfactory dysfunction in COVID-19: expression of ACE2, TMPRSS2, and Furin in the nose and olfactory bulb in human and mice	No inoculation
28	Yeast-Expressed SARS-CoV Recombinant Receptor-Binding Domain (RBD219-N1) Formulated with Alum Induces Protective Immunity and Reduces Immune Enhancement	SARS-CoV-1
29	Development of a COVID-19 vaccine based on the receptor binding domain displayed on virus-like particles	No inoculation
30	The WD and linker domains of ATG16L1 required for non-canonical autophagy limit lethal respiratory infection by influenza A virus at epithelial surfaces	Influenza A virus
31	SARS-CoV2 infection in farmed mink, Netherlands, April 2020	Cohort (not model)
32	Skin Delivery of Modified Vaccinia Ankara Viral Vectors Generates Superior T Cell Immunity Against a Respiratory Viral Challenge	Not SARS-CoV-2
33	Antibody repertoire induced by SARS-CoV-2 spike protein immunogens	No challenge
34	Expression of ACE2 and TMPRSS2 proteins in the upper and lower aerodigestive tracts of rats	No inoculation
35	Type III interferons disrupt the lung epithelial barrier upon viral recognition	Staphylococcus aureus
36	Type I and III interferons disrupt lung epithelial repair during recovery from viral infection	Influenza virus
37	Broad-spectrum antivirals of protoporphyrins inhibit the entry of highly pathogenic emerging viruses	Influenza virus
38	Global genetic patterns reveal host tropism versus cross-taxon	No inoculation

	transmission of bat Betacoronaviruses	
39	Individualized System for Augmenting Ventilator Efficacy (iSAVE): A Rapidly deployable system to expand ventilator capacity	No inoculation
40	Recombinant SARS-CoV-2 spike S1-Fc fusion protein induced high levels of neutralizing responses in nonhuman primates	No inoculation<br>Read in full
41	Single-cell atlas of a non-human primate reveals new pathogenic mechanisms of COVID-19	No inoculation
42	Structure, function and antigenicity of the SARS-CoV-2 spike glycoprotein	No inoculation
43	Susceptibility of tree shrew to SARS-CoV-2 infection	No disease model?
44	Therapeutic efficacy of Pudilan Xiaoyan Oral Liquid (PDL) for COVID-19 in vitro and in vivo	No methods
45	Prophylactic and therapeutic remdesivir (GS-5734) treatment in the rhesus macaque model of MERS-CoV infection	MERS-CoV
46	Chemical composition and pharmacological mechanism of Qingfei Paidu Decoction and Ma Xing Shi Gan Decoction against Coronavirus Disease 2019 (COVID-19): in silico and experimental study	LPS-PNEUMONIA
47	Immune modulation to improve survival of respiratory virus infections in mice	PNEUMONIA MODEL<br>NOT SARS CoV-2
48	Non-neuronal expression of SARS-CoV-2 entry genes in the olfactory system suggests mechanisms underlying COVID-19-associated anosmia	No inoculation
49	Immunoglobulin fragment F(ab')2 against RBD potently neutralizes SARS-CoV-2 in vitro	No inoculation
50	Glycomic analysis of host-response reveals high mannose as a key mediator of influenza severity	H1N1
51	Coronavirus surveillance of wildlife in the Lao People's Democratic Republic detects viral RNA in rodents	No inoculation
52	Mapping the Immunodominance Landscape of SARS-CoV-2 Spike Protein for the Design of Vaccines against COVID-19	No inoculation
53	Classical drug digitoxin inhibits influenza cytokine storm, with implications for COVID-19 therapy.	No inoculation
54	JAK1 inhibition blocks lethal sterile immune responses: implications for COVID-19 therapy	Viral mimetics
55	LY6E impairs coronavirus fusion and confers immune control of viral disease	MHV-A59 virus
56	Corona virus activates a stem cell mediated defense mechanism that accelerates activation of dormant tuberculosis: implications for the COVID-19 pandemic	MHV-1 virus
57	The SARS-CoV-2 receptor-binding domain elicits a potent neutralizing response without antibody-dependent enhancement	No inoculation
58	SARS-CoV-2 neutralizing serum antibodies in cats: a serological investigation	No inoculation

59	Absence of SARS-CoV-2 infection in cats and dogs in close contact with a cluster of COVID-19 patients in a veterinary campus	No inoculation
60	A single dose SARS-CoV-2 simulating particle vaccine induces potent neutralizing activities	Different viruses
61	Pericyte-specific vascular expression of SARS-CoV-2 receptor ACE2 – implications for microvascular inflammation and hypercoagulopathy in COVID-19 patients	No inoculation
62	Novel and potent inhibitors targeting DHODH, a rate-limiting enzyme in de novo pyrimidine biosynthesis, are broad-spectrum antiviral against RNA viruses including newly emerged coronavirus SARS-CoV-2	H1N1
63	Leveraging mRNAs sequences to express SARS-CoV-2 antigens in vivo	No inoculation
64	Indomethacin has a potent antiviral activity against SARS CoV-2 in vitro and canine coronavirus in vivo	Canine corona virus<br>Not SARS-CoV-2
65	Python nidoviruses, more than respiratory pathogens	Nidoviruses<br>Not SARS-CoV-2
66	Tissue-specific delivery system via AFF-1-coated pseudotyped Vesicular Stomatitis Virus in C. elegans	Not SARS-CoV-2
67	Non-invasive surveys of mammalian viruses using environmental DNA	No inoculation
68	Cellular senescence limits acute lung injury induced by mechanical ventilation	No inoculation
69	Are pangolins the intermediate host of the 2019 novel coronavirus (2019-nCoV) ?	No inoculation
70	Isolation and Characterization of 2019-nCoV-like Coronavirus from Malayan Pangolin	No inoculation
71	A novel bat coronavirus reveals natural insertions at the S1/S2 cleavage site of the Spike protein and a possible recombinant origin of HCoV-19	No inoculation
72	Mechanistic understanding enables the rational design of salicylanilide combination therapies for Gram-negative infections	Other organisms
	**CATEGORY C**	**Not original research**
**1**	The origin and underlying driving forces of the SARS-CoV-2 outbreak	Review
2	Severe acute respiratory syndrome-related coronavirus: The species and its viruses – a statement of the Coronavirus Study Group	Letter
3	Novel human coronavirus (SARS-CoV-2): A lesson from animal coronaviruses.	Review
4	COVID-19: Epidemiology, Evolution, and Cross-Disciplinary Perspectives	Review
5	Serum albumin-mediated strategy for the effective targeting of	Review

	SARS-CoV-2.	
6	Focus on Receptors for Coronaviruses with Special Reference to Angiotensin-converting Enzyme 2 as a Potential Drug Target - A Perspective.	Letter
7	Of mice and men: COVID-19 challenges translational neuroscience.	Letter
8	Does SARS-Cov-2 invade the brain? Translational lessons from animal models.	Review
9	Vaccines for SARS-CoV-2: Lessons from Other Coronavirus Strains.	Review
10	Is GSK3β a molecular target of chloroquine treatment against COVID-19?	Review
11	The anti-viral facet of anti-rheumatic drugs: Lessons from COVID-19.	Review
12	Cardiac Manifestations Of Coronavirus (COVID-19).	Review
13	[ACE-inhibitors, angiotensin receptor blockers and severe acute respiratory syndrome caused by coronavirus].	Review (Italian)
14	Should we expect neurological symptoms in the SARS-CoV-2 epidemic?	Review
15	Defining Protective Epitopes for COVID-19 Vaccination Models.	Letter
16	SARS-CoV-2 Vaccines: Status Report.	Report
17	ACE2 the Janus-faced protein - from cardiovascular protection to severe acute respiratory syndrome-coronavirus and COVID-19.	Letter
18	A consensus statement on the use of angiotensin receptor blockers and angiotensin converting enzyme inhibitors in relation to COVID-19 (corona virus disease 2019).	Letter
19	Perioperative Presentation of COVID-19 Disease in a Liver Transplant Recipient.	Case report
20	COVID-19 Challenges Basic Researchers	Letter
21	Don't rush to deploy COVID-19 vaccines and drugs without sufficient safety guarantees	Letter
22	Can the Coronavirus Disease 2019 (COVID-19) Affect the Eyes? A Review of Coronaviruses and Ocular Implications in Humans and Animals	Review
23	Compounds with Therapeutic Potential against Novel Respiratory 2019 Coronavirus	Review
24	Analysis of angiotensin-converting enzyme 2 (ACE2) from different species sheds some light on cross-species receptor usage of a novel coronavirus 2019-nCoV	letter
25	This scientist hopes to test coronavirus drugs on animals in locked-down Wuhan.	Letter
26	COVID-19 and the Heart	Review
27	SARS-CoV-2: Olfaction, Brain Infection, and the Urgent Need for Clinical Samples Allowing Earlier Virus Detection	Review
28	The possible mechanisms of action of 4-aminoquinolines (chloroquine/hydroxychloroquine) against Sars-Cov-2 infection	Review

	(COVID-19): A role for iron homeostasis?	
29	COVID-19 shot protects monkeys	Letter
30	Hypothesized behavioral host manipulation by SARS-CoV2/COVID-19 infection	Review
31	Animal models for emerging coronavirus: progress and new insights	Review
32	Kallikrein-kinin blockade in patients with COVID-19 to prevent acute respiratory distress syndrome	Review
33	Canine Respiratory Coronavirus: A Naturally Occurring Model of COVID-19?	Review
34	Reducing risks from coronavirus transmission in the home-the role of viral load	Review
35	Interactions between antihyperglycemic drugs and the renin-angiotensin system: Putative roles in COVID-19. A mini-review	Review
36	Development of Remdesivir Repositioning as a Nucleotide Analog Against COVID-19 RNA Dependent RNA Polymerase	Review
37	The renin-angiotensin system - a therapeutic target in COVID-19?	Review
38	Chaos theory applied to the outbreak of COVID-19: an ancillary approach to decision making in pandemic context	Review
39	Possible challenges in behavioral phenotyping of rodents following COVID-19 lockdown	Letter
40	Hypothesis for potential pathogenesis of SARS-CoV-2 infection–a review of immune changes in patients with viral pneumonia	Review
41	COVID-19, ACE2, and the cardiovascular consequences	Review
42	Zoonotic origins of human coronaviruses	Review
43	Coronaviruses and SARS-COV-2	Review
44	Learning from our immunological history: What can SARS-CoV teach us about SARS-CoV-2?	Letter
45	Interactions of coronaviruses with ACE2, angiotensin II, and RAS inhibitors-lessons from available evidence and insights into COVID-19	Review
46	Could Intravenous Immunoglobulin Collected from Recovered Coronavirus Patients Protect against COVID-19 and Strengthen the Immune System of New Patients?	Review
47	Covid-19, Coronavirus, SARS-CoV-2 and the small bowel	Review
48	First respiratory transmitted food borne outbreak?	Review
49	Is COVID-19 the first pandemic that evolves into a panzootic?	Review
50	The COVID-19 Pandemic: A Comprehensive Review of Taxonomy, Genetics, Epidemiology, Diagnosis, Treatment, and Control	Review
51	Harnessing innate immunity to eliminate SARS-CoV-2 and ameliorate COVID-19 disease	Letter
52	Sex-Specific SARS-CoV-2 Mortality: Among Hormone-Modulated ACE2 Expression, Risk of Venous Thromboembolism and Hypovitaminosis D	Review
53	Biological plausibility for interactions between dietary fat,	Review

	resveratrol, ACE2, and SARS-CoV illness severity	
54	Prevention and therapy of COVID-19 via exogenous estrogen treatment for both male and female patients	Review
55	Elevated exhaustion levels and reduced functional diversity of T cells in peripheral blood may predict severe progression in COVID-19 patients	Letter
56	Inhaled biguanides and mTOR inhibition for influenza and coronavirus (Review)	Review
57	A Review: Does Complement or the Contact System Have a Role in Protection or Pathogenesis of COVID-19?	Review
58	[SARS-CoV-2-a challenge for all of us]	Letter<br>German
59	COVID-19 Q&A: Animal care continues	Letter
60	Zoonotic origins of human coronavirus 2019 (HCoV-19 / SARS-CoV-2): why is this work important?	Letter
61	SARS-CoV2 induced respiratory distress: Can cannabinoids be added to anti-viral therapies to reduce lung inflammation?	Letter
62	COVID-19 Q&A: Keeping a cancer core going	Letter
